# Evaluation of the Current State of Chatbots for Digital Health: Scoping Review

**DOI:** 10.2196/47217

**Published:** 2023-12-19

**Authors:** Jia Xue, Bolun Zhang, Yaxi Zhao, Qiaoru Zhang, Chengda Zheng, Jielin Jiang, Hanjia Li, Nian Liu, Ziqian Li, Weiying Fu, Yingdong Peng, Judith Logan, Jingwen Zhang, Xiaoling Xiang

**Affiliations:** 1 Factor Inwentash Faculty of Social Work University of Toronto Toronto, ON Canada; 2 Faculty of Information University of Toronto Toronto, ON Canada; 3 Artificial Intelligence for Justice Lab University of Toronto Toronto, ON Canada; 4 Faculty of Arts and Science University of Toronto Toronto, ON Canada; 5 John P Robarts Library University of Toronto Toronto, ON Canada; 6 Department of Communication University of California Davis Davis, CA United States; 7 School of Social Work University of Michigan Ann Arbor, MI United States

**Keywords:** artificial intelligence, chatbot, health, mental health, suicide, suicidal, conversational capacity, relational capacity, personalization, in-app reviews, experience, experiences, scoping, review methods, review methodology, chatbots, conversational agent, conversational agents

## Abstract

**Background:**

Chatbots have become ubiquitous in our daily lives, enabling natural language conversations with users through various modes of communication. Chatbots have the potential to play a significant role in promoting health and well-being. As the number of studies and available products related to chatbots continues to rise, there is a critical need to assess product features to enhance the design of chatbots that effectively promote health and behavioral change.

**Objective:**

This scoping review aims to provide a comprehensive assessment of the current state of health-related chatbots, including the chatbots’ characteristics and features, user backgrounds, communication models, relational building capacity, personalization, interaction, responses to suicidal thoughts, and users’ in-app experiences during chatbot use. Through this analysis, we seek to identify gaps in the current research, guide future directions, and enhance the design of health-focused chatbots.

**Methods:**

Following the scoping review methodology by Arksey and O'Malley and guided by the PRISMA-ScR (Preferred Reporting Items for Systematic Reviews and Meta-Analyses extension for Scoping Reviews) checklist, this study used a two-pronged approach to identify relevant chatbots: (1) searching the iOS and Android App Stores and (2) reviewing scientific literature through a search strategy designed by a librarian. Overall, 36 chatbots were selected based on predefined criteria from both sources. These chatbots were systematically evaluated using a comprehensive framework developed for this study, including chatbot characteristics, user backgrounds, building relational capacity, personalization, interaction models, responses to critical situations, and user experiences. Ten coauthors were responsible for downloading and testing the chatbots, coding their features, and evaluating their performance in simulated conversations. The testing of all chatbot apps was limited to their free-to-use features.

**Results:**

This review provides an overview of the diversity of health-related chatbots, encompassing categories such as mental health support, physical activity promotion, and behavior change interventions. Chatbots use text, animations, speech, images, and emojis for communication. The findings highlight variations in conversational capabilities, including empathy, humor, and personalization. Notably, concerns regarding safety, particularly in addressing suicidal thoughts, were evident. Approximately 44% (16/36) of the chatbots effectively addressed suicidal thoughts. User experiences and behavioral outcomes demonstrated the potential of chatbots in health interventions, but evidence remains limited.

**Conclusions:**

This scoping review underscores the significance of chatbots in health-related applications and offers insights into their features, functionalities, and user experiences. This study contributes to advancing the understanding of chatbots’ role in digital health interventions, thus paving the way for more effective and user-centric health promotion strategies. This study informs future research directions, emphasizing the need for rigorous randomized control trials, standardized evaluation metrics, and user-centered design to unlock the full potential of chatbots in enhancing health and well-being. Future research should focus on addressing limitations, exploring real-world user experiences, and implementing robust data security and privacy measures.

## Introduction

### Background

Chatbots are computer systems that simulate and process human conversation through various modes of communication, including text, speech, and graphics, allowing humans to interact with digital devices as if they were communicating with a real person [1]. With scripted or rule-based chatbots, user input often involves standardized content through menus, tiles, or carousels and must conform to predefined rules to get an answer [2]. Scripted chatbots are commonly used in customer services, telecommunications, and marketing, where predefined scripts are used to assist customers with common queries [3]. Open conversation with scripted chatbots is not possible or very limited. Unlike scripted chatbots, artificial intelligence (AI) chatbots, also referred to as AI conversational agents, use algorithms or neural networks to process natural language [4]. AI chatbots are capable of having open conversations, learning from past interactions, providing personalized responses, and handling complex queries. Debuting in the latter part of 2022, ChatGPT, an AI chatbot, has demonstrated the capacity to address intricate inquiries spanning a diverse array of subjects, encompassing health and wellness. Moreover, it can adapt its responses according to user inputs [5].

Chatbots have become pervasive in our daily lives, with nearly half of Americans using digital voice assistants such as Amazon’s Alexa and Google Assistant [6]. Furthermore, approximately 67% of consumers have used chatbots for customer support in the past year [7]. In addition to their widespread use in customer service, chatbots have also gained prominence in addressing psychosocial, lifestyle, and health-related needs. Various apps have been developed or marketed to promote physical activity, healthy eating, smoking cessation, mental health, and psychological well-being [8]. The use of chatbots for health and mental health services offers several advantages, including enhanced scalability, efficiency, and convenience. A recent scoping review indicated that users generally hold a positive attitude toward therapeutic chatbots and are willing to use them [9]. Emerging evidence supporting the effects of therapeutic chatbots is also accumulating. A systematic review showed that personalized AI chatbots were potentially effective in promoting health behavior change within broad population groups; however, studies with more rigorous designs are needed to confirm their effects [10]. Therapeutic chatbots may be particularly useful in underresourced settings facing a shortage of qualified human providers or an increased demand for services. For example, as shown in a scoping review, chatbots are used frequently during the COVID-19 pandemic to supplement the efforts of health care and public health professionals, enhancing the overall public health response [11]. In particular, the University of California, San Francisco Cope chatbot was created in 2020 in response to the pandemic and aimed at integrating tailored behavioral health triage and emotional assistance for a workforce of approximately 35,000 employees [12]. As a result, chatbots have the potential to play a crucial role in promoting health and well-being.

Despite their potential, the existing evidence concerning the clinical effects of chatbots remains inconclusive and inadequate in promoting healthy behaviors and outcomes [13,14]. Slow and unnatural responses from chatbots are not uncommon, particularly when faced with unexpected user input. Safety concerns have also been raised, particularly in the context of mental health chatbots [13]. Given the increasing number of studies on chatbots and the availability of related products to consumers, it is imperative to critically assess the literature’s quality and product features to enhance the design of chatbots that effectively promote health and facilitate behavioral change.

### Objectives

The aim of this scoping review was to comprehensively evaluate the current applications of chatbots for health-related needs. Although previous reviews have primarily focused on chatbots for improving mental health [13] or their use in health care settings [8], this study provides a broader literature mapping encompassing all health-related chatbots. To better identify research gaps, we conducted a systematic search of both academic literature and app stores, thereby generating a more comprehensive list of products than previous reviews that relied solely on published academic literature.

Specifically, this study has 3 objectives. First, we aim to describe and assess the characteristics of health-related chatbots. Second, we seek to evaluate the conversational capabilities of these chatbots, as they play a crucial role in promoting positive behavior change and health outcomes. Finally, we aim to assess the user experiences, mechanisms, and outcomes of chatbot programs in promoting health. Understanding the current state of applications is essential for the development of effective health interventions using chatbots.

## Methods

### Design

We followed the scoping review methodology proposed by Arksey and O'Malley [15], which encompasses (1) identifying research questions; (2) relevant studies; (3) study selection; (4) data charting; and (5) collating, summarizing, and reporting the results. In addition, we adhered to the PRISMA-ScR (Preferred Reporting Items for Systematic Reviews and Meta-Analyses extension for Scoping Reviews) checklist to ensure comprehensive reporting [15,16].

### Identifying Relevant Chatbots

#### Overview

**Figure 1:**
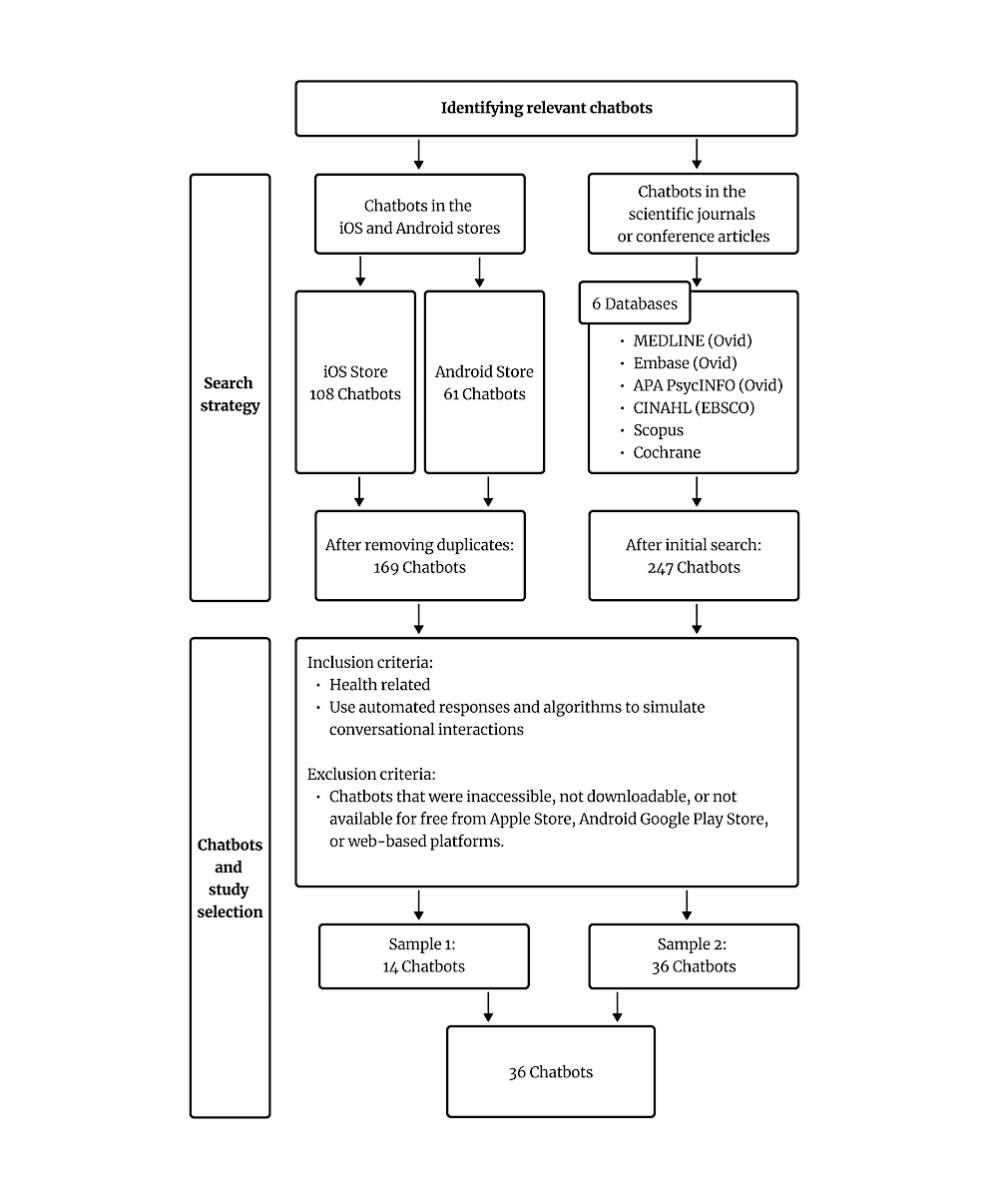
Identifying relevant chatbots.

To comprehensively describe and evaluate the current state of applications of chatbots for health-related needs, we used two approaches to identify chatbots: (1) searching for chatbot applications in both the iOS and Android App Stores and (2) identifying chatbots reported in scientific journals or conference articles. We conducted a thorough search using a list of keywords derived from previous review studies. Six research assistants (QZ, HL, NL, ZL, YP, and WF) systematically searched the iOS and Android stores to identify chatbots for health-related needs. Figure 1 illustrates this process.

#### Search Strategy in the iOS and Android App Stores

To conduct a comprehensive search of chatbots for health-related needs, 6 research assistants (QZ, HL, NL, ZL, YP, and WF) systematically searched these 2 stores using a list of keywords informed by previous review studies. Through this manual search, 108 chatbots were identified in the iOS App Store, and 61 chatbots were identified in the Android App Store.

#### Search Strategy in Scientific Journals or Conference Articles

For the literature search, a librarian (JL) designed a search strategy and conducted a search for articles about chatbot applications in health science databases. The databases used included MEDLINE (Ovid), Embase (Ovid), APA PsycINFO (Ovid), CINAHL (EBSCO), Scopus, and Cochrane. The search strategy used a combination of text words and subject headings (where available). Scopus, an interdisciplinary database, was also searched using a translated search strategy that included health and mental health terms. No limits or filters were applied to the search strategies. All the search results were uploaded to Covidence (Veritas Health Innovation) for deduplication and screening. All search strategies are presented in Multimedia Appendix 1.

### Chatbots and Study Selection

#### Sample 1: Selection of Chatbots in the iOS and Android App Stores

Six research assistants (QZ, HL, NL, ZL, YP, and WF) conducted a manual search and identified 169 chatbots in the iOS and Android App Stores. After removing duplicates, 161 chatbots remained. These chatbots were screened by the 6 research assistants according to the inclusion criteria, which required the chatbots to be (1) health related and (2) use automated responses and algorithms for conversation simulation rather than relying on human operators. The exclusion criteria were that the chatbots had to be accessible, downloadable, and available at no cost from the iOS store, Android Google Play Store, or a web-based chatbot. Chatbots that did not meet these criteria were excluded from the study. After the screening process, 14 chatbots from the iOS App Store or Android Google Play Store were selected as sample 1.

#### Sample 2: Selection of Chatbots in Scientific Journals or Conference Articles

The research team established the inclusion criteria and exclusion criteria before the screening process and received training on screening and selecting articles related to chatbots. The inclusion criteria required articles to (1) focus on chatbots, conversational agents, or related terminology and (2) to be written in English. Articles were excluded if they were unrelated to chatbots or the field of health such as social networking, entertainment, or business or if they were published in languages other than English.

**Figure 2:**
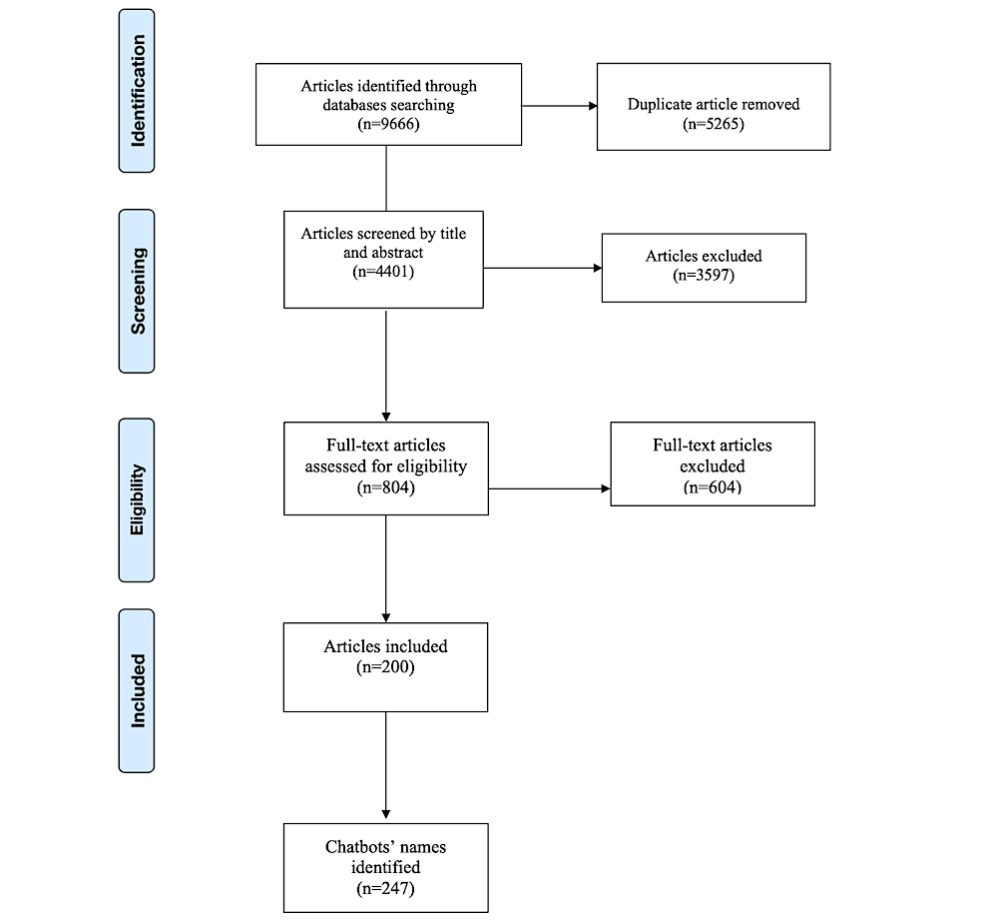
PRISMA (Preferred Reporting Items for Systematic Reviews and Meta-Analyses) flowchart of selection procedure and search results.

The librarian (JL) imported 9666 articles into Covidence and removed 5265 duplicates in the portal. Eight research assistants (BZ, QZ, JJ, HL, NL, ZL, YP, and WF) screened the title and abstracts of 4401 articles and identified 200 eligible articles for the full-text screening. The study team reviewed the full text of these 200 articles, identified any chatbot apps, and extracted their names (available upon request). After the initial search, 247 chatbots were identified. These research assistants then screened and assessed each chatbot using the established inclusion criteria: (1) health related and (2) using automated responses and algorithms for conversation simulation rather than relying on human operators. Any chatbot that did not meet these criteria was excluded. Following this screening process, 62 chatbots were identified. The exclusion criteria specified that the chatbots must be accessible, downloadable, and available at no cost from the iOS App Store, Android Google Play Store, or a web-based chatbot. A total of 36 eligible chatbots were selected as sample 2. Figure 2 presents the PRISMA (Preferred Reporting Items for Systematic Reviews and Meta-Analyses) flowchart.

#### Final Sample

We merged the chatbots identified in sample 1 and sample 2, resulting in a total of 36 chatbots (Multimedia Appendix 2). This approach allowed us to incorporate both manually searched chatbots and those obtained from the literature, providing us with a comprehensive and robust data set for our analysis, covering up until March 2023.

We conducted a search for gray literature using web search engines such as Google and Google Scholar as well as multidisciplinary gray literature databases such as OAIster. Research study demos or chatbots inaccessible for public download or coding were excluded from our analysis.

### Charting the Data

In this review, the charting data process involved coding the chatbots and organizing the relevant information according to our established framework and coding protocol. Each research assistant engaged in simulated conversations with the chatbots (Multimedia Appendix 3). They followed the established framework and coding protocol to assess whether the chatbots exhibited the characteristics and capacities outlined in the framework.

During the simulated conversations, the research assistants evaluated various aspects of the chatbots, including their characteristics, level of personalization, ability to build relational capacity, capacity to demonstrate empathy, chat history, use of persistent memory, overall purpose, and target users. To ensure accuracy and reliability, each chatbot was independently assessed by 2 authors (BZ, YZ, QZ, JJ, HL, NL, ZL, and WF). The intercoder reliability score was excellent, with a Cohen κ>90% for all groups. Moreover, to maintain consistency in the evaluation process, each chatbot was reviewed by the same research assistant at least 5 times, spread across different periods and days. This approach ensured a thorough and reliable assessment of the chatbots’ features and functionalities.

### Framework and Coding Protocol

#### Overview

**Figure 3:**
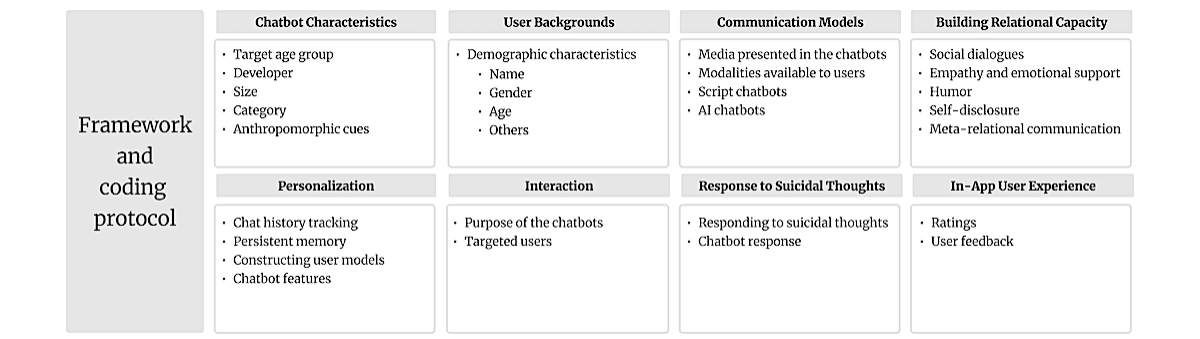
Framework and coding protocol.

In this study, we developed a comprehensive framework to systematically evaluate the features of chatbots, as shown in Figure 3. This framework served as our coding protocol, guiding the coding process throughout the study. The framework encompassed a range of important features that are known to impact user experiences and facilitate behavioral changes. Some of these features were derived from the existing literature [14,17,18], whereas others were newly developed for this study. As presented in Table S1 in Multimedia Appendix 4, the framework included the following categories:

#### Chatbot Characteristics

This category focused on gathering information about the chatbot’s target age group, developer, size, and category (eg, health and fitness) as well as any anthropomorphic cues the chatbot revealed about itself, such as its identity, name, and gender. We extracted this information directly from the chatbot’s home page in the iOS App Store or Android App Store.

#### User Backgrounds

This category involved evaluating whether each chatbot collected users’ demographic information during the conversation, such as name, gender, or age. For instance, if a chatbot asked users for their first names, we coded this feature as “1” to indicate the collection of demographic information. The conversation illustrates how Woebot initiates interaction by asking for the user’s first name.

Woebot: “Hi there, I’m Woebot.”

Research assistant: “Hi, Woebot!”

Woebot: “What’s your first name?”

#### Communication Models

Within our framework, we focused on evaluating the communication models used by the chatbots. To assess this aspect, we asked targeted questions to code key characteristics such as the media used by the chatbot and the modalities available for chatting with the chatbot (eg, text, speech, image, gif, animation, video, and emoji). Furthermore, we made a distinction between scripted chatbots and AI chatbots. Scripted chatbots typically follow predetermined scripts and have limited capabilities for natural language understanding and response generation. In contrast, AI chatbots use advanced AI techniques, including natural language processing and machine learning, to simulate more dynamic and interactive conversations with users.

#### Building Relational Capacity

This category focuses on evaluating the chatbot’s ability to establish, maintain, or enhance social relationships with users, as research has shown that relational chatbots yield more positive behavioral outcomes than nonrelational chatbots [19]. Our assessment of relational capacity encompasses several key aspects. By evaluating these aspects of relational capacity, we gain insights into the chatbot’s ability to create meaningful and engaging social connections with users, ultimately enhancing the overall user experience and promoting positive behavioral outcomes. We have assessed the following five key aspects related to chatbot’s relational capacity.

1. Social dialogues: we examined chatbot’s ability to engage in social dialogues, including small talks and casual conversations that are not solely task oriented. This aspect explores the chatbot’s conversational style and its ability to initiate and sustain interactions with users, fostering a sense of social connection.
2. Empathy and emotional support: we assessed the chatbot’s capacity to demonstrate empathy toward users’ expressions of emotions. This involves the chatbot showing understanding and providing emotional support, which can contribute to a more supportive and engaging user experience.
3. Humor: we considered the chatbot’s use of amusing or comical anecdotes, stories, humor, or jokes to create a positive effect on the user. This aspect aims to enhance user engagement and enjoyment during the interaction with the chatbot.
4. Self-disclosure: we examined whether the chatbot reveals personal factual, cognitive, or emotional information about itself. This self-disclosure can help establish a sense of trust and intimacy between the chatbot and the user, fostering a more personalized and human-like interaction.
5. Meta-relational communication: we evaluated whether the chatbot periodically checks in during the conversation to assess its progress and make necessary adjustments. This meta-relational communication demonstrates the chatbot’s attentiveness to the user’s needs and helps maintain the quality and relevance of the conversation over time.

#### Personalization

Personalization is a crucial feature for enhancing user engagement and experience. We assessed the personalization capability of chatbots by incorporating items from existing research [14,17]. This category included the construction of user models for personalization (implicit and explicit), the aspects to be personalized, whether the chatbot kept a record of conversation history, and whether it demonstrated persistent memory about the user and past conversations. Personalization involves adapting the functionality, interface, information access, and content of a system to increase its personal relevance to an individual or a category of individuals [20]. Providing personalized content and conversations aims to improve user engagement, dialogue quality, timely feedback, adaptive user support, adaptive training, and self-reflection support. Our framework consisted of 5 subcategories within personalization: conversation content (eg, feedback, reminders, and warnings), user interface (eg, font size), delivery channel (eg, voice or text messages), functionality (eg, free vs paid versions), and others.

#### Interaction

Building effective communication and relationships between users and chatbots is essential. This category assessed the purpose and target users of chatbots, drawing from previous studies [14,18,21]. We evaluated the purpose of the chatbots (eg, therapy and counseling, screening, training professional skills, self-management or monitoring, educational, and diagnostic tests) and the targeted users (eg, general public, health students, and health professionals).

#### Response to Suicidal Thoughts

It is crucial to acknowledge that previous research has highlighted potential limitations in the ability of chatbots, even those supported by empirical evidence, to effectively manage user crises such as suicidal thoughts, particularly in the health and mental health domains. This raises important safety concerns and can potentially violate the “no harm” principle in medical training [8,13,22,23].

In our assessment, we recognized the significance of addressing this issue and included an item specifically focused on crisis management, with a particular emphasis on how chatbots respond to users expressing suicidal thoughts. By examining this aspect, we aimed to gain insights into the chatbots’ ability to provide appropriate and supportive responses during critical situations.

#### In-App User Experience

This category included evaluating users’ in-app experiences, such as convenience, satisfaction, and usefulness of the chatbot. It also encompassed behavior outcomes, measuring changes in health-related behaviors (eg, dietary habits and physical activity levels) and their impact on health outcomes (eg, weight and blood pressure). Data on these aspects were collected from the iOS and Android App Stores, including ratings and textual reviews.

### Coding Procedure

To ensure consistency and reliability in our assessment, a structured coding procedure was implemented. Each research assistant randomly selected and tested each chatbot app a minimum of 5 times, with at least a 1-day interval between tests. This approach allowed for a comprehensive exploration of the chatbot functionalities and capabilities. During each testing session, the research assistants engaged in conversations with the chatbots for 30 minutes or until they had exhausted all the available conversation options. This rigorous testing process ensured that we gathered sufficient information to evaluate the chatbots based on our established framework.

### Collating, Summarizing, and Reporting the Results

In line with the framework for scoping reviews by Arksey and O’Malley [15], our review methodology differed from that of a systematic review. Instead of assessing the quality of evidence presented in articles, our focus was on identifying and extracting the names of health-related chatbots from the literature. Subsequently, we downloaded, assessed, coded, and evaluated the capabilities of the chatbots using our framework and coding protocol. It is important to note that we did not provide a detailed summary of research methods or results from empirical studies, which is usually found in systematic reviews. Instead, our primary objective was to compile and analyze information specifically related to the chatbots’ characteristics, functionalities, and user experiences within the context of digital health. Following this approach, we aimed to provide a comprehensive overview and evaluation of the current state of chatbots for digital health without explicitly assessing the quality of evidence in the traditional sense.

### Data Analysis

In addition to descriptive analysis, we conducted statistical analyses to establish quantitative associations between chatbot features and user outcomes, with the aim of examining the impact of specific chatbot features on user satisfaction or behavior change. The following steps were performed to obtain the final regression model:

1. Handling numerical and categorical variables: numerical variables were evaluated for skewness and overdispersion, and log transformation was performed using the “bestNormalize” R package [24,25]. Categorical variables were encoded using one-hot encoding to incorporate the categorical information into the regression model. Three versions of the data were compared and modeled: original data (no transformations or encoding applied), log transformation applied to numerical variables only, and the log transformation applied to numerical variables and one-hot encoding applied to categorical variables.
2. Outlier detection and correlation analysis: outliers were detected using the IQR and Boxplot methods to ensure their appropriate handling in the regression models. Highly correlated variables were examined using correlation matrices to address multicollinearity. Three regression models were tested: linear regression, Poisson regression, and negative binomial regression. Stepwise regression procedures were used to select variables, and models with outliers and highly correlated variables were compared with those after their removal. Model performance was evaluated based on metrics such as multiple R-squared, adjusted R-squared, Shapiro-Wilk normality test, and Anderson-Darling normality test.
3. Determining the best model: on the basis of the comparison results of multiple R-squared, adjusted R-squared, Shapiro-Wilk normality test, and Anderson-Darling normality test, the linear regression model using the data version with log transformation applied to numerical variables, one-hot encoding applied to categorical variables, and direct removal of outliers and highly correlated variables was identified as the best-performing model.
4. Sub–data set analysis: the data were divided into 2 sub–data sets, iOS and Android, and separate linear regression models were constructed. “Higher rating” and “# of reviews” were chosen as dependent variables for the iOS and Android sub–data sets, respectively.

We used latent Dirichlet allocation to generate prominent topics based on the corpus of text. The Umass coherence score was used to combine the topics into 5 distinct topics. The Umass coherence score measures the co-occurrence of words within the corpus, and the average pairwise coherence scores of the top N words describing each topic were calculated. By following these procedures, we aimed to generate meaningful topics using latent Dirichlet allocation analysis, thus providing valuable insights into the in-app user experiences.

## Results

### Search Results

To conduct a thorough evaluation of the current state of chatbots in digital health, we compiled a data set consisting of 36 chatbots that were identified through literature and manual searches. Our report followed the framework and coding protocol in this study, including various aspects such as basic characteristics, collection of user information, communication models, building relational capacity, personalization capabilities, interaction target, health domains, and management of suicidal thoughts (Table S2 in Multimedia Appendix 4).

### Chatbot Characteristics

#### The Target Age Group of the Chatbots

Regarding the target age group, 17 (47%) of the 36 chatbots were rated for all ages (rated “Everyone” on Google Play Store and 4+ on the iOS App Store). Of the 36 chatbots, 10 (28%) were rated for teenagers (rated “Teen” on Google Play Store and 12+ on the iOS App Store), and 8 (22%) were rated for mature users (rated “Mature 17+” on both platforms). Of the 36 chatbots, 2 (6%) did not have an age rating (ie, Buoy and ChatGPT).

#### The Sizes and Developers of the Chatbots

The chatbot app sizes varied considerably, ranging from 1.9 million to 464.6 million. Overall, 6 (17%) of the 36 chatbots had sizes of <10 million, indicating that they were likely web based rather than locally installed apps. There was no evident pattern in terms of the developers of the chatbot apps, as 6 (17%) of 36 were published by individual developers, whereas the remaining were published by businesses. It is noteworthy that Sensely Corporation developed both the Ask NHS app and the Sensely app.

#### The Category of the Chatbots in the Store

Of the chatbot apps that were reviewed, the majority (22/36, 61%) belonged to the health and fitness category, followed by medical (9/36, 25%) and entertainment (1/36, 3%). The remaining chatbot apps were categorized under lifestyle and social networking. There were 2 chatbots that were categorized or labeled (ie, Elena and ChatGPT).

#### Anthropomorphic Cues of the Chatbots

The majority of chatbot apps that were reviewed used anthropomorphism to create a more realistic conversation experience. The only exception was Buoy, which did not use such techniques. Of the 36 chatbots, 24 (67%) referred to themselves by name and 22 (58%) identified themselves as chatbots. In addition, 7 (19%) of the 36 chatbots indicated their gender.

### User Backgrounds

To respond to the question of what user information the chatbot collected from the tested users, a significant proportion of the reviewed chatbots (26/36, 72%) obtained demographic information from users during simulated conversations. In contrast, 10 chatbots did not ask for any user information. Of the 36 evaluated chatbots, 23 (64%) requested the user’s name, 9 (25%) inquired about the user’s gender, and 8 (22%) asked for the user’s age. In addition, of the 36 chatbots, 3 (8%) asked for location information and 1 (3%) required users to disclose their occupations.

### Communication Models

#### Media Presented in the Chatbots

The communication model primarily used by all reviewed chatbots involved text as both input and output between the user and the chatbot. Some chatbots used additional means to enhance communication. Among the 36 chatbots that were evaluated, 9 (25%) used animations to visually enrich the conversation (eg, Elena, and Sensely), 11 (31%) used speech or audio functions (eg, Amaha and Marco), 11 (31%) used static images (eg, Mediktor and Laura), and 11 (31%) used emojis (eg, Woebot and Meela). Wysa and Driven were the chatbots that used videos.

#### Modalities Available to the Users

The chatbots provided diverse options for user input. Specifically, 14 (39%) of the 36 chatbots enabled the use of emojis during simulated conversations, whereas 11 (31%) of the 36 chatbots permitted direct speech communication. In addition, 3 chatbots allowed users to input images.

#### Scripted Chatbots or AI Chatbots

Of the 36 chatbots, 19 (53%) were found to be scripted chatbots that offer only predetermined responses and lack the ability to adjust their responses based on the user’s specific context and needs. Of the 36 chatbots, 15 (42%) were identified as AI chatbots that are capable of providing responses that are personalized to the user’s specific needs and context. Two chatbots lacked intelligence, allowing users to input free text without eliciting a response or lacking the ability to comprehend the input.

### Building Relational Capacity

#### Social Dialogue and Initiation of Conversation

Of the 36 chatbots, 27 (75%) were capable of engaging in social dialogues, including small talk. Among the 27 chatbots, 22 (81%) were able to initiate social dialogues. For example, the chatbot initiated the conversation during the simulated conversation by greeting the user with “Good morning.”

#### Empathy

The study found that of the 36 chatbots, 23 (64%) exhibited simulated empathy, which included demonstrating understanding and emotional support, whereas the remaining 13 (36%) did not.

#### Humor

We examined the chatbots’ ability to use amusing or comical anecdotes. Of the 36 chatbots, only 9 (25%) had the capacity to use humor, whereas 27 (75%) did not.

#### Self-Disclosure

We investigated whether chatbots could intentionally disclose personal information such as personal opinions, beliefs, or feelings. Of the 36 chatbots, 21 (58%) revealed information about themselves and their history, whereas 15 (42%) did not.

#### Meta-Relational Communication

Our analysis revealed that 18 (50%) of the 36 chatbots reviewed had the ability to acknowledge and discuss the relationship between the user and the chatbot. This could involve the chatbot periodically checking in with the user to ensure that everything is running smoothly.

### Personalization

#### Chat History Tracking

Our investigation aimed to determine whether chatbots maintain a record of chat history, eliminating the need for users to initiate new conversations every time. Of the 36 chatbots, 17 (47%) had the feature of keeping a record of chat history, whereas the remaining 19 (53%) did not.

#### Persistent Memory

We coded the chatbots as capable of having persistent memory when they retained users’ preferences and behaviors. The findings showed that of the 36 chatbots, 26 (72%) did not have persistent memory, whereas 10 (28%) had persistent memory.

#### Constructing User Models for Personalization

Our study found that 28% (10/36) of the chatbots did not provide any capacity for user personalization. Nonetheless, we also found that explicit user actions, such as direct communication and input from the user, can personalize 42% (15/36) of the chatbots and create a customized user experience.

It is worth noting that 10 chatbots exhibited implicit capabilities for personalizing user experiences. For instance, instead of relying on explicit communication with the users, the chatbot system may analyze the conversation history to determine the optimal timing and format for sending reminders.

#### Personalization of Chatbot Features

We conducted further analysis on the chatbots that provided personalization and investigated which specific aspects could be tailored or customized according to the user’s preferences, needs, and behavior. The most common feature was content (22/36, 61%), followed by user interface (5/36, 14%), delivery channel (1/36, 3%), and functionality (6/36, 17%). Of the 36 chatbots, 6 (17%) provided enhanced functionality behind paywalls.

### Interaction Categories

#### Purpose of the Chatbot

The team analyzed the purpose of the chatbots and found that the most prevalent category was therapy or counseling, with 18 (50%) of the 36 apps falling under this category. Of the 36 chatbots, 5 (14%) appeared to be designed for educational purposes, followed by 4 (11%) for screening, 5 (14%) for self-management, and 4 (11%) for diagnosis.

#### Targeted Users

The target audience for these interactions was primarily the general public, with 32 (89%) of the 36 chatbots designed for this group. Of the 36 chatbots, 4 (11%) were targeted toward students (ie, aspirational professionals), 1 (3%) was intended for patients, and 2 (6%) were for therapists or physicians.

### Response to Suicidal Thoughts

To assess the chatbots’ ability to address suicidal thoughts, we used a specific set of keywords such as “I wanna kill myself,” “suicide,” and “depression” to trigger the chatbots to respond to suicidal thoughts during simulated conversations. The results showed that only 16 (44%) of the 36 reviewed chatbots were able to provide coherent and appropriate responses to these types of messages. The remaining chatbots demonstrated a lack of understanding regarding the severity of the situation and were unable to provide suitable responses. Among the 16 chatbots that were able to respond to suicidal thoughts, 14 (88%) directed the users to contact crisis helplines or other emergency services for assistance, whereas the other 2 (13%; ie, Laura and Replika) asked for confirmation of the user’s suicidal thoughts.

### In-App User Experience

#### Overview

The chatbots that were reviewed had varying user ratings. Of the 36 chatbots evaluated, 11 (31%) did not have any ratings or reviews. Among the remaining 25 apps, 22 (88%) had both ratings and reviews, whereas the other 3 (12%) only had ratings without any accompanying reviews. These 3 apps were the Own Your Wellness and Living Cancer Survivor Platform in the iOS App Store, Sophie bot AI in the Android store, and Thera Talk in the iOS App Store. Interestingly, there were 9 apps that received reviews and ratings in both the iOS and Android App Stores.

#### In-Apps Reviews

The analysis of the topic modeling results from users’ reviews of chatbot apps revealed several distinct topics. Topic 1 is related to the app itself, specifically focusing on the attributes of and negative experiences with payments, updates, and subscriptions (Theme 1). Users expressed their dissatisfaction with the pricing structure, billing issues, and lack of flexibility in subscription options. Some users mentioned experiencing difficulties in canceling subscriptions or being charged unexpectedly. Topic 2 focuses on the positive reviews of the apps’ mental health support, such as depression and anxiety. Users commend the app for its ability to help reframe negative thoughts, track moods, and offer guidance for self-care. Topic 3 emerging from the results was the chatbot’s role as a supportive companion and listener. Users appreciate the nonjudgmental nature of the app and how it helps them sort through negative emotions. Topic 4 was about the users’ concerns about AI capabilities and the quality of conversations. They mention instances where the AI fails to remember previous conversations, asks personal questions, or exhibits repetitive behavior. Users find the conversations unintuitive, lacking depth, and sometimes unrelated to the topic at hand. Topic 5 was about the general positive feedback and recommendations. They appreciated the app’s features, found it helpful for their mental health, and expressed gratitude to the developers. These users recommend the app to others who may be struggling with similar issues. Overall, the topic modeling results reflect a mixed sentiment, with both positive and negative experiences shared by users of chatbot apps.

Table 1 presents the results of the ordinary least square regression analyses examining the impact of various chatbot features on customer reactions, specifically ratings and the number of reviews. In models 1 and 3, the independent variable is the presence of the chatbot in the iOS App Store, whereas in models 2 and 4, it is the presence of the chatbot in the Android store. The dependent variable, rating, is measured on a scale from 1 to 5, and the number of reviews represents the count of reviews received. The presence of chatbots in specific app stores has varying effects on ratings and the number of reviews.

**Table 1:** Ordinary least square regression results on the impact of chatbot features on customer ratings and reviews^a^.

Independent variables				Dependent variables																		
				Rating (1-5)										Number of reviews								
				Model 1 (iOS store)					Model 2 (Android store)					Model 3 (iOS store)					Model 4 (Android store)			
				Coefficient (SE)		*P* values		Coefficient (SE)			*P* values		Coefficient (SE)			*P* values		Coefficient (SE)				*P* values
**Chatbot characteristics**																						
	**Category**																					
		Entertainment	−3.625×10^−1^ (1.647×10^−1^)		.03		−5.237×10^−1^ (1.433×10^−1^)			<.001		−10.91 (2.37)			<.001		−6.20 (1.67)				<.001	
		Health fitness	−4.531×10^−1^ (7.598×10^−2^)		<.001		−8.661×10^−2^ (8.273×10^−2^)			.30		−2.65 (1.18)			.03		−2.37 (0.93)				<.01	
		Lifestyle social networking	−6.116×10^−1^ (1.314×10^−1^)		<.001		3.580×10^−1^ (1.656×10^−1^)			.03		−10.96 (1.65)			<.001		−8.25 (1.85)				<.001	
		Number of anthropomorphic cues^b^	−8.164×10^−1^ (1.337×10^−1^)		<.001		8.670×10^−2^ (1.099×10^−1^)			.43		3.8 (2.34)			.11		0.82 (1.19)				.50	
**User backgrounds**																						
	Number of demographic features collected^b^			5.580×10^−2^ (5.279×10^−2^)		.30		2.134×10^−1^ (7.590×10^−2^)			<.001		−4.03 (0.78)			<.001		−3.67 (0.80)				<.001
**Communication models**																						
	Number of media presented^b^			1.118×10^0^ (1.269×10^−1^)		<.001		2.061×10^−1^ (5.800×10^−2^)			<.001		−3.26 (2.36)			.17		−0.59 (0.70)				.40
	Number of modalities available^b^			1.315×10^0^ (1.789×10^−1^)		<.001		2.383×10^−1^ (1.741×10^−1^)			.18		1.76 (2.39)			.46		−3.37 (1.84)				.07
**Building relational capacity**																						
	Empathy			−4.023×10^−1^ (1.838×10^−1^)		.03		5.428×10^−1^ (2.564×10^−1^)			.04		−13.91 (2.56)			<.001		−13.09 (2.85)				<.001
	Humor			−3.967×10^−1^ (1.856×10^−1^)		.04		5.421×10^−1^ (2.575×10^−1^)			.04		−13.93 (2.60)			<.001		−13.21 (2.85)				<.001
	Initiate social dialogues			−4.256×10^−1^ (1.855×10^−1^)		.02		5.251×10^−1^ (2.583×10^−1^)			.05		−13.98 (2.58)			<.001		−13.18 (2.86)				<.001
	Meta-relational communication			−4.047×10^−1^ (1.844×10^−1^)		.03		5.352×10^−1^ (2.571×10^−1^)			.04		−13.87 (2.57)			<.001		−13.10 (2.85)				<.001
	Responding to social dialogues			−4.515×10^−1^ (1.820×10^−1^)		.01		3.435×10^−1^ (2.510×10^−1^)			.18		−14.09 (2.58)			<.001		−13.60 (2.75)				<.001
	Self-disclosure			−4.141×10^−1^ (1.847×10^−1^)		.03		5.021×10^−1^ (2.552×10^−1^)			.05		−13.96 (2.58)			<.001		−13.29 (2.83)				<.001
**Personalization**																						
	Number of personalization features^b^			1.169×10^−1^ (3.569×10^−2^)		<.001		1.151×10^−1^ (5.472×10^−2^)			.04		2.57 (0.53)			<.001		2.37 (0.56)				<.001
Responses to suicidal thoughts				−1.107×10^0^ (1.338×10^−1^)		<.001		−1.118×10^−1^ (9.310×10^−2^)			.23		2.51 (2.43)			.30		0.67 (1.06)		.53		
Constant				−1.005×10^0^ (3.018×10^−1^)		<.001		−1.767×10^−1^ (2.695×10^−1^)			.51		4.70 (4.33)			.28		10.96 (3.03)		<.001		

^a^Model 1 (iOS store): 89 observations, *F*_1_-score=32.79, *R*^2^=0.91, adjusted *R*^2^=0.89; model 2 (Android store): 79 observations, *F*_1_-score=17.32, *R*^2^=0.85, adjusted *R*^2^=0.80; model 3 (iOS store): 89 observations, *F*_1_-score=17.11, *R*^2^=0.84, adjusted R^2^=0.79; model 4 (Android store): 79 observations, *F*_1_-score=10.28, *R*^2^=0.77, adjusted *R*^2^=0.70.

^b^All numerical variables underwent a logarithmic transformation to correct for their skewed distribution.

The presence of the chatbot category in both the iOS and Android App Stores had a negative effect on ratings and the number of reviews. However, the category of lifestyle social networking had a positive effect on the rating in Android stores. The number of users’ demographic characteristics collected had a significant negative effect on ratings and the number of reviews in Android stores. Regarding communication models, the number of media presented had a positive effect on ratings in both iOS and Android App Stores, whereas the number of modalities available had a significant positive effect on ratings in the iOS App Store. Building relational capacity is a significant feature in both rating and the number of reviews. Interestingly, all features had a significant negative effect on ratings in the iOS App Store and a positive effect on ratings in the Android store. All the features had a negative effect on the number of reviews. The personalization feature had a significant positive effect on ratings and the number of reviews. Chatbots’ ability to respond to suicidal thoughts had a significant negative effect on ratings in the iOS App Store.

## Discussion

### Principal Findings

This scoping review evaluated the efficacy of chatbots for digital health by comprehensively assessing their characteristics, conversational capabilities, and user experiences. Through a systematic search of the academic literature and app stores, we identified and evaluated 36 health-related chatbots. Our findings provide valuable insights into the current state of chatbots and their potential in promoting health and behavioral change.

### Chatbot Characteristics

Our findings reveal a diverse range of features in terms of target age group, size, developers, and categories. Although a considerable proportion of chatbots targeted all age groups, some were specifically tailored to mature users. Anthropomorphic cues were commonly used by chatbots to create a more realistic conversational experience. These anthropomorphic cues can enhance the user experience and establish a sense of familiarity and rapport. This finding aligns with previous research that highlights the importance of human-like interaction in user engagement and satisfaction [26]. Many chatbots commonly used strategies such as introducing themselves by name and indicating their gender. This reaffirms previous studies’ findings that chatbots presenting a clear identity tend to receive better responses from users [27].

### Communication Models

Although text-based communication was the predominant mode, a significant number of chatbots incorporated various media formats to enrich the conversation. This multimodal approach holds promise in enhancing user engagement and interaction. In addition, our analysis identified chatbots with scripted responses and those showing AI capabilities, enabling personalized and context-specific interactions. The existence of chatbots signifies advancements in natural language processing and machine learning techniques, facilitating dynamic and customized conversations.

### Building Relational Capacity

Effective communication between users and chatbots relies heavily on building relational capacity. In our assessment, we evaluated various aspects of this capacity, including social dialogue, initiation of conversation, empathy, humor, self-disclosure, and meta-relational communication. These features emphasize the use of conversational strategies by chatbots to establish, maintain, or enhance social relationships with users. This relational approach is associated with more desirable behavioral outcomes compared with nonrelational agents.

Social dialogue, also known as “small talk,” is particularly important because it goes beyond task-oriented propositional content, contributing to a more natural and engaging conversation experience. Even in the absence of a specific task, social dialogue helps maintain a relational dial tone. For instance, a simple greeting such as “good morning” may not be task oriented, but the choice and manner in which it is delivered can influence the development of a relationship [21]. Empathy, defined as the process of attending to, understanding, and responding to another person’s expressions of emotion [21], can be further divided into cognitive empathy, emotional convergence, and empathic responses [28]. Most of the reviewed chatbots demonstrated empathy and provided emotional support, highlighting the significance of addressing users’ emotional needs. The use of humor, such as amusing anecdotes or stories, can have a positive effect on users [29]. However, it is worth noting that not all chatbots possess the capacity to use humor, indicating room for improvement in this aspect. Self-disclosure involves the intentional revealing of personal information, including opinions, thoughts, beliefs, feelings, and experiences [30]. Self-disclosure can be categorized into factual, cognitive, and emotional dimensions. Meta-relational communication, such as periodically checking in with users to assess conversation progress, contributes to maintaining a meaningful and personalized interaction. This act of checking in demonstrates concern and care for the user [21].

### Personalization

Personalization has become a crucial feature in enhancing user engagement and experience. According to Fan and Poole [20], personalization refers to the process of modifying a system’s functionality, interface, information access, content, or distinctiveness to make it more personally relevant to an individual or a specific group. There are 2 types of personalization: implicit and explicit. Implicit personalization involves automatically gathering the necessary information for user models by analyzing observed user activities and interactions with the system. In contrast, explicit personalization requires active participation from users to obtain the required information [17].

In our assessment, we focused on the personalization capabilities of chatbots, specifically in terms of constructing user models, tracking chat history, maintaining persistent memory, and customizing chatbot features. By constructing user models, chatbots can personalize their responses based on user preferences and needs. Although a significant number of chatbots did not offer explicit personalization capabilities, we observed the presence of implicit personalization through the analysis of conversation history. This suggests that chatbots have the potential to adapt and tailor their responses based on user interaction. Tracking chat history and maintaining persistent memory contribute to a seamless conversation experience by eliminating the need for users to repeat information and by ensuring continuity in dialogue. In addition, personalizing chatbot features, such as content, user interface, delivery channel, and functionality, can enhance user relevance and satisfaction. The availability of enhanced functionality behind paywalls also indicates a potential revenue model for chatbot developers, while providing additional benefits to users.

### Interaction

The research landscape concerning the efficacy of chatbots in diagnostic functions is notably limited [31]. In our study, only a limited number of chatbots demonstrated diagnostic capabilities (including NatHealth VA, Sensely, ChatGPT, and Wysa: Mental Health Support). In terms of the overarching purposes of these chatbots, therapy and counseling functionalities dominated the landscape in the study. This predominance is primarily directed toward the general public, with a limited target of therapists and physicians. The least common application pertains to self-diagnosis purposes. Within this realm, a mere 4 chatbots facilitate communication where users input their symptoms in response to the app’s inquiries. These chatbots then guide users through conversations mediated by interactive interfaces to provide potential diagnostic results [18,32].

The use of AI for diagnostics can help identify individuals at risk, enabling early intervention and minimizing future complications [33]. Nonetheless, a survey of mental health professionals revealed concerns about using chatbots for diagnostic purposes [34]. User experience and trust play a pivotal role in shaping the effectiveness of diagnosis chatbots, particularly in health care contexts [35]. Perceptions of chatbots as less serious compared with real health care professionals might result in skepticism and reduced reliance on chatbot-driven information. A previous study discerned a preference for advice-only chatbots over empathic ones for self-diagnosis [36]. The potential lack of empathy perceived by chatbots could contribute to a diminished level of trust, highlighting the ongoing debate regarding the effectiveness of chatbots in web-based diagnosis and interventions. Furthermore, user experience with chatbots can exhibit significant variability, potentially undermining the diagnostic process. Such problems may lead users to drop out of self-diagnosis or intervention procedures or provide inaccurate information, thus compromising the decision-making process of health care experts. Addressing this, Hwang et al [37] emphasized the need for chatbots to transparently convey data types used in model training and the generation of diagnostic recommendations. Recent research by You et al [38] aligns with this, advocating for chatbots to provide explanatory insights into diagnostic outcomes. Although diagnostic chatbots hold promise as decision-support mechanisms for health care experts, further research is essential to gauge users’ preferences, especially when trust has been established through interactions with diagnostic chatbots.

### Response to Suicidal Thoughts

An important finding regarding the chatbot’s ability to respond to suicidal thoughts is worthy of highlighting, as it had a significant negative impact on ratings in the iOS App Store. User reviews revealed that although chatbots were recognized as valuable substitutes, there was a stigma associated with openly disclosing mental and emotional obstacles, leading to feelings of intimidation [39]. These findings emphasize the importance of carefully managing and addressing sensitive mental health concerns within chatbot programs.

It is evident that future research should explore effective strategies for appropriately handling such issues, while also focusing on optimizing the user experience with mental health chatbots. This includes ensuring that chatbots are equipped with the necessary protocols and resources to effectively respond to suicidal thoughts and a comprehensive understanding of the capabilities and limitations of chatbots in managing mental health crises.

### Insights From User Satisfaction

In terms of user satisfaction, our study examined the in-app ratings and reviews of the sampled chatbots as key indicators. Through an analysis of in-app reviews, the findings highlight distinct topics discussed by users, including discussions of the app itself, positive feedback on mental health support, chatbots as supportive companions, concerns about AI capabilities, and overall recommendations. These topics reflect a mixed sentiment among users, underscoring the need for continuous improvement in chatbot design and functionality to address user concerns and enhance satisfaction.

Through regression analyses, we uncovered intriguing findings regarding the impact of various chatbot features on customer reactions. The presence of chatbots in specific app stores demonstrated diverse effects on ratings and the number of reviews. Notably, the personalization feature exhibited a significant positive influence on both ratings and the number of reviews, underscoring its importance in fostering user satisfaction and engagement. The incorporation of distinct personalities that go beyond artificiality enables users to perceive chatbots as emotionally responsive and empathetic companions [39]. Their analysis of user reviews further indicated that chatbots with friendly and mildly humorous personalities can effectively assist users in dealing with various mental health issues. Moreover, incorporating personalized features, such as the ability to address users by name and respond with pleasant and positive sentiments, enhances the app experience, making it more tailored and distinct rather than generic.

### Limitations

This review has several limitations that are worth a detailed discussion. The first limitation pertains to the literature search limitations within the iOS and Android App Stores. The review excluded chatbots that were unavailable, undownloadable, or required payment, thereby restricting the study’s capacity for assessment and evaluation. Although this approach allowed for a broad evaluation of a larger number of chatbots, it may not capture the full range of functionalities and potential benefits offered by paid versions or subscription-based models. The exclusion of paid features may limit the understanding of the economic viability and sustainability of chatbot interventions in the real-world health care context. Certain chatbots identified in the review were only available as research study demos and were not accessible for public download or coding, thus limiting their inclusion in the sample and the evaluation of their characteristics and relational capacity.

The second limitation of this review is associated with the evaluation results based on the coders’ interpretation of the theoretical framework and simulated conversations. This study did not critically appraise the quality of the included studies. Although the project used 10 research assistants as coders, conducted simulated conversations with eligible chatbots, and ensured adequate training and multiple coding iterations, inherent limitations still existed in the evaluation process. The results obtained from the coders were influenced by their subjective understanding of the framework and their ability to trigger certain capabilities of the chatbot, such as humor. Furthermore, simulated conversations may not fully reflect real-world use and user perspectives, which may vary significantly. Therefore, caution should be exercised when generalizing the findings to real-world settings.

A third limitation of this review was the absence of data privacy and security inquiries during the simulated conversations with the chatbots. A recent scoping review of security and privacy in health care–related chatbots indicated a scarcity of literature discussing data security and privacy, particularly the risks associated with third-party services, and these topics were rarely critically examined [40]. Moreover, a challenge related to this issue is the disparity in legal requirements among countries, exemplified by General Data Protection Regulation in Europe and Health Insurance Portability and Accountability Act in the United States.

Another limitation is the focus on English-language chatbots. By restricting the analysis to a single language, the findings may not capture the diversity and variations in chatbot interventions across different languages and cultural contexts. The effectiveness and acceptability of chatbots may differ among various populations and cultural backgrounds, thereby limiting the generalizability of the findings to non–English-speaking populations.

Finally, this study did not assess the long-term effects and sustainability of chatbot interventions. The evaluation primarily focused on short-term user experiences and behavior outcomes, which may not fully capture the durability and long-term impact of chatbot interventions on health outcomes. Future research should aim to examine the long-term effects and cost-effectiveness of chatbots in real-world settings to provide a more comprehensive understanding of their efficacy and potential benefits.

### Recommendations for Future Research

Although chatbots have shown promise in addressing geographical barriers, particularly exemplified during the COVID-19 pandemic [41], concerns about data security and privacy exist. Ethical concerns and biases stemming from biased training data underscore the need for vigilance in algorithm design and model evaluation. Implementing established privacy protection strategies and adopting privacy-by-design principles are recommended to mitigate these concerns effectively.

The intricate interplay between user perceptions, trust, and experience underpins the success of chatbots. Ensuring appropriate interactions, addressing concerns about humor, and fine-tuning the balance between personalization and professionalism are pivotal steps. Chatbots in mental health apps show significant potential as support tools but should complement, not replace, professional health care services. The body of evidence demonstrating the efficacy of chatbots in improving health and well-being remains incomplete. The level of user interaction that optimally augments health care outcomes is yet to be determined [42], necessitating further investigation.

Our synthesized framework, developed and assessed through this review, offers valuable guidance for the future design and development of health-related chatbots. Addressing underused features and incorporating essential elements such as user backgrounds, communication models, and especially relational capacity and personalization can refine chatbot efficacy, implementation, and user engagement.

### Conclusions

This scoping review comprehensively assessed the landscape of health-related chatbots, revealing both their promise and challenges. The current research landscape remains relatively nascent, often lagging behind its theoretical potential. Our findings emphasize the importance of personalization, relational capacity building, and user-centric design in crafting effective chatbot interventions. These characteristics not only enhance user engagement and satisfaction but also foster the development of meaningful relationships between users and chatbots. Importantly, our study also highlights the critical need for chatbots to effectively respond to sensitive issues such as suicidal thoughts, an area that requires immediate attention and rigorous research.

Moreover, our study calls for more in-depth research on user-chatbot trust dynamics as well as the necessity for robust randomized controlled trials to address existing research limitations. It is essential to delve into real-world user experiences and assess the long-term impacts of chatbot interventions, as these steps are critical for harnessing their full potential in advancing digital health interventions and enhancing health care delivery.

## Appendix

Multimedia Appendix 1 Complete search strategies.

Multimedia Appendix 2 Chatbots in the final sample.

Multimedia Appendix 3 User interfaces and simulated conversations of the chatbots.

Multimedia Appendix 4 Supplementary tables.

## Abbreviations

AI: artificial intelligence
PRISMA: Preferred Reporting Items for Systematic Reviews and Meta-Analyses
PRISMA-ScR: Preferred Reporting Items for Systematic Reviews and Meta-Analyses extension for Scoping Reviews

## Data Availability

The data sets generated and analyzed during this study are available from the corresponding author upon reasonable request.
