# Supplementary material for: Evaluation of the Current State of Chatbots for Digital Health: Scoping Review
Source: J Med Internet Res. 2023 Dec 19;25:e47217. doi: 10.2196/47217 (PMC10762606; doi:10.2196/47217)
Supplement: Multimedia Appendix 1 [file jmir_v25i1e47217_app1.docx]

# Appendix: Complete search strategies

## Ovid MEDLINE: Epub Ahead of Print, In-Process & Other Non-Indexed Citations, Ovid MEDLINE® Daily and Ovid MEDLINE®

1946-Present

| # | Searches | Results |
| --- | --- | --- |
| 1 | (Animated character*1 or Artificial agent*1 or Artificial* intelligen* or Assistant chat program*1 or Automated agent*1 or Avatar*1 or Chat agent*1 or Chat assistant*1 or Chat bot or Chat bots or Chatbot*1 or Chatter bot or Chatter bots or Chatter box or Chatter boxes or Chatterbot*1 or Chatterbox*2 or Communicative agent*1 or Companion agent*1 or Companion assistant*1 or Conversational agent*1 or Conversational AI or Conversational assistant*1 or Conversational avatar*1 or Conversational bot or Conversational bots or Conversational character*1 or Conversational computer*1 or Conversational entit* or Conversational human* or Conversational interface*1 or Conversational personal assistant*1 or Conversational system*1 or Conversational user interface*1 or Counsel* agent*1 or Cyber individual*1 or Dialog system*1 or Dialogue system*1 or Digital assistant*1 or Digital coach* or Embodied agent*1 or Intelligent agent*1 or Interactive agent*1 or Interactive online character*1 or Interactive talking program*1 or Interface agent*1 or Interface character*1 or Knowbot*1 or Motivational agent*1 or Online chat program*1 or Pedagogical agent*1 or Persuasive ECA or Relational agent*1 or Relational assistant*1 or Smart bot or Smart bots or Smartbot*1 or Socia* agent*1 or Socia* bot or Socia* bots or Soft bot or Soft bots or Softbot*1 or Software agent*1 or Talk bot or Talk bots or Talkbot*1 or Talking agent*1 or Talking avatar*1 or Text-based synchronous chat or Virtual advisor*1 or Virtual agent*1 or Virtual assistant*1 or Virtual character*1 or Virtual chat expert*1 or Virtual coach* or Virtual consultant*1 or Virtual counsel* or Virtual health agent*1 or Virtual health coach* or Virtual health counsel* or Virtual host*1 or Virtual human* or Virtual online assistant*1 or Virtual patient advocate*1 or Virtual personal assistant*1 or Virtual therapist*1 or Virtual web assistant*1).tw,kf. | 15165 |
| 2 | Artificial intelligence/ | 24065 |
| 3 | 1 or 2 | 36042 |
| 4 | (app or apps).tw,kf. | 30565 |
| 5 | ((Mobile or smartphone or tablet or device) adj1 application*1).tw,kf. | 7894 |
| 6 | Mobile applications/ | 6611 |
| 7 | 4 or 5 or 6 | 38410 |
| 8 | 3 and 7 | 360 |

## Embase (Ovid)

1947 to 2020 December 14

| # | Searches | Results |
| --- | --- | --- |
| 1 | (Animated character*1 or Artificial agent*1 or Artificial* intelligen* or Assistant chat program*1 or Automated agent*1 or Avatar*1 or Chat agent*1 or Chat assistant*1 or Chat bot or Chat bots or Chatbot*1 or Chatter bot or Chatter bots or Chatter box or Chatter boxes or Chatterbot*1 or Chatterbox*2 or Communicative agent*1 or Companion agent*1 or Companion assistant*1 or Conversational agent*1 or Conversational AI or Conversational assistant*1 or Conversational avatar*1 or Conversational bot or Conversational bots or Conversational character*1 or Conversational computer*1 or Conversational entit* or Conversational human* or Conversational interface*1 or Conversational personal assistant*1 or Conversational system*1 or Conversational user interface*1 or Counsel* agent*1 or Cyber individual*1 or Dialog system*1 or Dialogue system*1 or Digital assistant*1 or Digital coach* or Embodied agent*1 or Intelligent agent*1 or Interactive agent*1 or Interactive online character*1 or Interactive talking program*1 or Interface agent*1 or Interface character*1 or Knowbot*1 or Motivational agent*1 or Online chat program*1 or Pedagogical agent*1 or Persuasive ECA or Relational agent*1 or Relational assistant*1 or Smart bot or Smart bots or Smartbot*1 or Socia* agent*1 or Socia* bot or Socia* bots or Soft bot or Soft bots or Softbot*1 or Software agent*1 or Talk bot or Talk bots or Talkbot*1 or Talking agent*1 or Talking avatar*1 or Text-based synchronous chat or Virtual advisor*1 or Virtual agent*1 or Virtual assistant*1 or Virtual character*1 or Virtual chat expert*1 or Virtual coach* or Virtual consultant*1 or Virtual counsel* or Virtual health agent*1 or Virtual health coach* or Virtual health counsel* or Virtual host*1 or Virtual human* or Virtual online assistant*1 or Virtual patient advocate*1 or Virtual personal assistant*1 or Virtual therapist*1 or Virtual web assistant*1).tw,kw. | 19608 |
| 2 | Artificial intelligence/ | 28086 |
| 3 | 1 or 2 | 36813 |
| 4 | (app or apps).tw,kw. | 42661 |
| 5 | ((Mobile or smartphone or tablet or device) adj1 application*1).tw,kw. | 9352 |
| 6 | Mobile applications/ | 11433 |
| 7 | 4 or 5 or 6 | 52594 |
| 8 | 3 and 7 | 596 |

## APA PsycInfo (Ovid)

1806 to December Week 1 2020

| # | Searches | Results |
| --- | --- | --- |
| 1 | (Animated character*1 or Artificial agent*1 or Artificial* intelligen* or Assistant chat program*1 or Automated agent*1 or Avatar*1 or Chat agent*1 or Chat assistant*1 or Chat bot or Chat bots or Chatbot*1 or Chatter bot or Chatter bots or Chatter box or Chatter boxes or Chatterbot*1 or Chatterbox*2 or Communicative agent*1 or Companion agent*1 or Companion assistant*1 or Conversational agent*1 or Conversational AI or Conversational assistant*1 or Conversational avatar*1 or Conversational bot or Conversational bots or Conversational character*1 or Conversational computer*1 or Conversational entit* or Conversational human* or Conversational interface*1 or Conversational personal assistant*1 or Conversational system*1 or Conversational user interface*1 or Counsel* agent*1 or Cyber individual*1 or Dialog system*1 or Dialogue system*1 or Digital assistant*1 or Digital coach* or Embodied agent*1 or Intelligent agent*1 or Interactive agent*1 or Interactive online character*1 or Interactive talking program*1 or Interface agent*1 or Interface character*1 or Knowbot*1 or Motivational agent*1 or Online chat program*1 or Pedagogical agent*1 or Persuasive ECA or Relational agent*1 or Relational assistant*1 or Smart bot or Smart bots or Smartbot*1 or Socia* agent*1 or Socia* bot or Socia* bots or Soft bot or Soft bots or Softbot*1 or Software agent*1 or Talk bot or Talk bots or Talkbot*1 or Talking agent*1 or Talking avatar*1 or Text-based synchronous chat or Virtual advisor*1 or Virtual agent*1 or Virtual assistant*1 or Virtual character*1 or Virtual chat expert*1 or Virtual coach* or Virtual consultant*1 or Virtual counsel* or Virtual health agent*1 or Virtual health coach* or Virtual health counsel* or Virtual host*1 or Virtual human* or Virtual online assistant*1 or Virtual patient advocate*1 or Virtual personal assistant*1 or Virtual therapist*1 or Virtual web assistant*1).tw. | 10939 |
| 2 | Artificial intelligence/ | 8549 |
| 3 | 1 or 2 | 15521 |
| 4 | (app or apps).tw. | 7634 |
| 5 | ((Mobile or smartphone or tablet or device) adj1 application*1).tw. | 1739 |
| 6 | Mobile applications/ | 1013 |
| 7 | 4 or 5 or 6 | 8765 |
| 8 | 3 and 7 | 97 |

## CINAHL (EBSCO)

Inception to December 16, 2020

| # | Query | Results |
| --- | --- | --- |
| S7 | S3 AND S6 | 285 |
| S6 | S4 OR S5 | 16,060 |
| S5 | MH "mobile applications" | 8,116 |
| S4 | TI ( app OR apps ) OR AB ( app OR apps ) OR TI ( (mobile OR smartphone OR tablet OR device) N1 application* ) OR AB ( (mobile OR smartphone OR tablet OR device) N1 application* ) | 12,459 |
| S3 | S1 OR S2 | 8,142 |
| S2 | MH "Artificial intelligence" OR MH Knowbots | 4,509 |
| S1 | TI ( "Animated character*" OR "Artificial agent*" OR "Artificial* intelligen*" OR "Assistant chat program*" OR "Automated agent*" OR Avatar* OR "Chat agent*" OR "Chat assistant*" OR "Chat bot" OR "Chat bots" OR Chatbot* OR "Chatter bot" OR "Chatter bots" OR "Chatter box" OR "Chatter boxes" OR Chatterbot* OR Chatterbox* OR "Communicative agent*" OR "Companion agent*" OR "Companion assistant*" OR "Conversational agent*" OR "Conversational AI" OR "Conversational assistant*" OR "Conversational avatar*" OR "Conversational bot" OR "Conversational bots" OR "Conversational character*" OR "Conversational computer*" OR "Conversational entit*" OR "Conversational human*" OR "Conversational interface*" OR "Conversational personal assistant*" OR "Conversational system*" OR "Conversational user interface*" OR "Counsel* agent*" OR "Cyber individual*" OR "Dialog system*" OR "Dialogue system*" OR "Digital assistant*" OR "Digital coach*" OR "Embodied agent*" OR "Intelligent agent*" OR "Interactive agent*" OR "Interactive online character*" OR "Interactive talking program*" OR "Interface agent*" OR "Interface character*" OR Knowbot* OR "Motivational agent*" OR "Online chat program*" OR "Pedagogical agent*" OR "Persuasive ECA" OR "Relational agent*" OR "Relational assistant*" OR "Smart bot" OR "Smart bots" OR Smartbot* OR "Socia* agent*" OR "Socia* bot" OR "Socia* bots" OR "Soft bot" OR "Soft bots" OR Softbot* OR "Software agent*" OR "Talk bot" OR "Talk bots" OR Talkbot* OR "Talking agent*" OR "Talking avatar*" OR "Text-based synchronous chat" OR "Virtual advisor*" OR "Virtual agent*" OR "Virtual assistant*" OR "Virtual character*" OR "Virtual chat expert*" OR "Virtual coach*" OR "Virtual consultant*" OR "Virtual counsel*" OR "Virtual health agent*" OR "Virtual health coach*" OR "Virtual health counsel*" OR "Virtual host*" OR "Virtual human*" OR "Virtual online assistant*" OR "Virtual patient advocate*" OR "Virtual personal assistant*" OR "Virtual therapist*" OR "Virtual web assistant*" ) OR AB ( "Animated character*" OR "Artificial agent*" OR "Artificial* intelligen*" OR "Assistant chat program*" OR "Automated agent*" OR Avatar* OR "Chat agent*" OR "Chat assistant*" OR "Chat bot" OR "Chat bots" OR Chatbot* OR "Chatter bot" OR "Chatter bots" OR "Chatter box" OR "Chatter boxes" OR Chatterbot* OR Chatterbox* OR "Communicative agent*" OR "Companion agent*" OR "Companion assistant*" OR "Conversational agent*" OR "Conversational AI" OR "Conversational assistant*" OR "Conversational avatar*" OR "Conversational bot" OR "Conversational bots" OR "Conversational character*" OR "Conversational computer*" OR "Conversational entit*" OR "Conversational human*" OR "Conversational interface*" OR "Conversational personal assistant*" OR "Conversational system*" OR "Conversational user interface*" OR "Counsel* agent*" OR "Cyber individual*" OR "Dialog system*" OR "Dialogue system*" OR "Digital assistant*" OR "Digital coach*" OR "Embodied agent*" OR "Intelligent agent*" OR "Interactive agent*" OR "Interactive online character*" OR "Interactive talking program*" OR "Interface agent*" OR "Interface character*" OR Knowbot* OR "Motivational agent*" OR "Online chat program*" OR "Pedagogical agent*" OR "Persuasive ECA" OR "Relational agent*" OR "Relational assistant*" OR "Smart bot" OR "Smart bots" OR Smartbot* OR "Socia* agent*" OR "Socia* bot" OR "Socia* bots" OR "Soft bot" OR "Soft bots" OR Softbot* OR "Software agent*" OR "Talk bot" OR "Talk bots" OR Talkbot* OR "Talking agent*" OR "Talking avatar*" OR "Text-based synchronous chat" OR "Virtual advisor*" OR "Virtual agent*" OR "Virtual assistant*" OR "Virtual character*" OR "Virtual chat expert*" OR "Virtual coach*" OR "Virtual consultant*" OR "Virtual counsel*" OR "Virtual health agent*" OR "Virtual health coach*" OR "Virtual health counsel*" OR "Virtual host*" OR "Virtual human*" OR "Virtual online assistant*" OR "Virtual patient advocate*" OR "Virtual personal assistant*" OR "Virtual therapist*" OR "Virtual web assistant*" ) | 5,247 |

## Cochrane Central Register of Controlled Trials

Issue 12 of 12, December 2020

"Animated character*" OR "Artificial agent*" OR "Artificial* intelligen*" OR "Assistant chat program*" OR "Automated agent*" OR avatar* OR "Chat agent*" OR "Chat assistant*" OR "Chat bot" OR "Chat bots" OR chatbot* OR "Chatter bot" OR "Chatter bots" OR "Chatter box" OR "Chatter boxes" OR chatterbot* OR chatterbox* OR "Communicative agent*" OR "Companion agent*" OR "Companion assistant*" OR "Conversational agent*" OR "Conversational AI" OR "Conversational assistant*" OR "Conversational avatar*" OR "Conversational bot" OR "Conversational bots" OR "Conversational character*" OR "Conversational computer*" OR "Conversational entit*" OR "Conversational human*" OR "Conversational interface*" OR "Conversational personal assistant*" OR "Conversational system*" OR "Conversational user interface*" OR "Counsel* agent*" OR "Cyber individual*" OR "Dialog system*" OR "Dialogue system*" OR "Digital assistant*" OR "Digital coach*" OR "Embodied agent*" OR "Intelligent agent*" OR "Interactive agent*" OR "Interactive online character*" OR "Interactive talking program*" OR "Interface agent*" OR "Interface character*" OR knowbot* OR "Motivational agent*" OR "Online chat program*" OR "Pedagogical agent*" OR "Persuasive ECA" OR "Relational agent*" OR "Relational assistant*" OR "Smart bot" OR "Smart bots" OR smartbot* OR "Socia* agent*" OR "Socia* bot" OR "Socia* bots" OR "Soft bot" OR "Soft bots" OR softbot* OR "Software agent*" OR "Talk bot" OR "Talk bots" OR talkbot* OR "Talking agent*" OR "Talking avatar*" OR "Text-based synchronous chat" OR "Virtual advisor*" OR "Virtual agent*" OR "Virtual assistant*" OR "Virtual character*" OR "Virtual chat expert*" OR "Virtual coach*" OR "Virtual consultant*" OR "Virtual counsel*" OR "Virtual health agent*" OR "Virtual health coach*" OR "Virtual health counsel*" OR "Virtual host*" OR "Virtual human*" OR "Virtual online assistant*" OR "Virtual patient advocate*" OR "Virtual personal assistant*" OR "Virtual therapist*" OR "Virtual web assistant*" in Title Abstract Keyword AND app OR apps OR ( ( mobile OR smartphone OR tablet OR device ) NEAR/1 application* ) in Title Abstract Keyword

## Scopus

Inception to December 16, 2020

( TITLE-ABS-KEY ( "Animated character*" OR "Artificial agent*" OR "Artificial* intelligen*" OR "Assistant chat program*" OR "Automated agent*" OR avatar* OR "Chat agent*" OR "Chat assistant*" OR "Chat bot" OR "Chat bots" OR chatbot* OR "Chatter bot" OR "Chatter bots" OR "Chatter box" OR "Chatter boxes" OR chatterbot* OR chatterbox* OR "Communicative agent*" OR "Companion agent*" OR "Companion assistant*" OR "Conversational agent*" OR "Conversational AI" OR "Conversational assistant*" OR "Conversational avatar*" OR "Conversational bot" OR "Conversational bots" OR "Conversational character*" OR "Conversational computer*" OR "Conversational entit*" OR "Conversational human*" OR "Conversational interface*" OR "Conversational personal assistant*" OR "Conversational system*" OR "Conversational user interface*" OR "Counsel* agent*" OR "Cyber individual*" OR "Dialog system*" OR "Dialogue system*" OR "Digital assistant*" OR "Digital coach*" OR "Embodied agent*" OR "Intelligent agent*" OR "Interactive agent*" OR "Interactive online character*" OR "Interactive talking program*" OR "Interface agent*" OR "Interface character*" OR knowbot* OR "Motivational agent*" OR "Online chat program*" OR "Pedagogical agent*" OR "Persuasive ECA" OR "Relational agent*" OR "Relational assistant*" OR "Smart bot" OR "Smart bots" OR smartbot* OR "Socia* agent*" OR "Socia* bot" OR "Socia* bots" OR "Soft bot" OR "Soft bots" OR softbot* OR "Software agent*" OR "Talk bot" OR "Talk bots" OR talkbot* OR "Talking agent*" OR "Talking avatar*" OR "Text-based synchronous chat" OR "Virtual advisor*" OR "Virtual agent*" OR "Virtual assistant*" OR "Virtual character*" OR "Virtual chat expert*" OR "Virtual coach*" OR "Virtual consultant*" OR "Virtual counsel*" OR "Virtual health agent*" OR "Virtual health coach*" OR "Virtual health counsel*" OR "Virtual host*" OR "Virtual human*" OR "Virtual online assistant*" OR "Virtual patient advocate*" OR "Virtual personal assistant*" OR "Virtual therapist*" OR "Virtual web assistant*" ) ) AND ( TITLE-ABS-KEY ( app OR apps OR ( ( mobile OR smartphone OR tablet OR device ) W/1 application* ) ) ) AND ( TITLE-ABS-KEY ( "Case work*" OR casework* OR client* OR clinic* OR counsel* OR diagnos* OR disease* OR disorder* OR doctor* OR ehealth OR "e-health" OR health* OR illness* OR medical* OR mental* OR mhealth OR "M-Health" OR nurs* OR patient* OR physician* OR psycholog* OR psychotherap* OR "Social work*" OR telediagnos* OR telehealth* OR telemedicine OR therap* OR treatment* OR "Well being" OR wellbeing OR wellness ) )
