# Supplementary material for: Evaluation of the Current State of Chatbots for Digital Health: Scoping Review
Source: J Med Internet Res. 2023 Dec 19;25:e47217. doi: 10.2196/47217 (PMC10762606; doi:10.2196/47217)
Supplement: Multimedia Appendix 2 [file jmir_v25i1e47217_app2.docx]

**Appendix 2. Chatbots in the final sample**

|  | **Chatbots Name** | **iOS**  **Store** | **Android Store** | **Website Service** | **Developer** | **Developer’ Address** | **Developer’ Country** | **Developer’ Continent** |
| --- | --- | --- | --- | --- | --- | --- | --- | --- |
| 1 | Amaha: Mental Health Self-Care / Amaha (InnerHour): self-care | **✓** | **✓** |  | InnerHour | Amaha, 5th Floor, WeWork, Raheja Platinum, Marol CHS Rd, off Andheri - Kurla Road, Marol, Andheri East, Mumbai - 400059 | India | South Asia |
| 2 | Anesthesia Assistant | **✓** |  |  | Faizan Ullah | Invotyx Suite 3 52 Upton Lane, London, United Kingdom, E7 9LN +44 07958 137157 | United Kingdom | Northwestern Europe |
| 3 | AskFirst (formerly Ask NHS) | **✓** | **✓** |  | Sensely Corporation | Primary Office 1871 26th Avenue San Francisco, CA 94122 United States | United States | North America |
| 4 | Buoy Health |  |  | **✓** | Buoy Health | 580 Harrison Ave, Boston, MA 02118, United States | United States | North America |
| 5 | Chat Bot |  | **✓** |  | Kings Dev | N/A | N/A | N/A |
| 6 | ChatGPT |  |  | **✓** | OpenAI | 3180 18th St, San Francisco, California, 94110, United States | United States | North America |
| 7 | ChatPal |  | **✓** |  | Cork Institute of Technology | Rossa Ave, Bishopstown, Cork, T12 P928, Ireland | Ireland | North-western Europe |
| 8 | Coco Nutritionist | **✓** |  |  | James Glass | Marcia Davidson  Spoken Language Systems  MIT Computer Science and Artificial Intelligence Laboratory  32 Vassar Street 32-G434  Cambridge, MA 02139 USA | United States | North America |
| 9 | Driven Resilience App | **✓** | **✓** |  | [Hello Driven](https://play.google.com/store/apps/developer?id=Hello+Driven) | 9 Castlereagh St, Sydney NSW 2000, Australia | Australia | Australia |
| 10 | Elena-The Medical Assistant |  | **✓** |  | Skealy Studios | Shozhinganallur, Chennai, 600119 | India | South Asia |
| 11 | Elysai: Talk to AI Friends |  | **✓** |  | [PromethistAI a.s.](https://play.google.com/store/apps/dev?id=5442656805872185667) | PromethistAI a. s. Salvátorská 931/8 110 00 Praha 1 | Czechia | Central Europe |
| 12 | IWill Care | **✓** |  |  | Epsyclinic | Gurugram, India, Haryana | India | South Asia |
| 13 | Laura | **✓** | **✓** |  | Tilde SIA | Vienības gat. 75a, Zemgales priekšpilsēta, Rīga, LV-1004, Latvia | Latvia | Northern Europe |
| 14 | Magnify Wellness | **✓** |  |  | Abigayle Peterson | Western Washington University | United States | North America |
| 15 | MARCo: Mental Health Friend | **✓** |  |  | Marco Technologies | 15260 Commerce Dr S, Dearborn, MI 48120, United States | United States | North America |
| 16 | MayaMD | **✓** | **✓** |  | [Maya MD, Inc](https://apps.apple.com/ca/developer/maya-md-inc/id1453357821) | 745 Atlantic Ave., 8th Floor Boston, MA | United States | North America |
| 17 | Mediktor - Symptom Checker | **✓** |  |  | Teckel Medical s.l. | Sant Antoni Maria Claret 167, 08025 Barcelona Barcelona Spain | Spain | Southern Europe |
| 18 | Meela - Birth support and more | **✓** |  |  | [Juno Technology, LLC](https://play.google.com/store/apps/developer?id=Juno+Technology,+LLC) | 195 Stanton St, New York, NY 10002, USA | United States | North America |
| 19 | NatHealth VA | **✓** |  |  | [National Health Insurance Admin. Co. (NatHealth)](https://play.google.com/store/apps/developer?id=National+Health+Insurance+Admin.+Co.+(NatHealth)) | Rafiq Al Hariri Ave 14, Amman | Jordan | Middle East |
| 20 | Naturopathy Assistant |  |  |  | [Vijayakumar P](https://play.google.com/store/apps/dev?id=8307895920269035680) | Sardar Patel Road Anna University Chennai - 600 025 | India | South Asia |
| 21 | OWL Cancer Survivor Platform | **✓** |  |  | Stayhealthy | 800 Royal Oaks Dr. Suite 102 Monrovia CA, 91016 | United States | North America |
| 22 | PocketFriend Digital Therapy | **✓** |  |  | Jason Gallate | The University of Sydney, NSW 2006  Australia | Australia | Australia |
| 23 | Reflectly - Journal & AI Diary / Reflectly: Mood Tracker Diary | **✓** |  |  | [Reflectly ApS](https://apps.apple.com/us/developer/reflectly-aps/id469957278) | Balticagade 14B 8000 Aarhus C Denmark | Denmark | Northern Europe |
| 24 | Replika - Virtual AI Companion / Replika: My AI Friend | **✓** |  |  | [Luka, Inc](https://play.google.com/store/apps/developer?id=Luka,+Inc) | 1266 Harrison St San Francisco, CA 94103 United States | United States | North America |
| 25 | RiseUp: AI based CBT Therapy | **✓** |  |  | [Gopal Goyal](https://apps.apple.com/gb/developer/gopal-goyal/id1636269124) | Mahendragarh, Haryana, India | India | South Asia |
| 26 | Sensely | **✓** |  |  | [Sensely Corporation](https://play.google.com/store/apps/developer?id=Sensely+Corporation) | 229 Kearny St, San Francisco, CA 94108, USA | United States | North America |
| 27 | SophieBot AI |  |  |  | [STAND UP SHOUT OUT](https://play.google.com/store/apps/developer?id=STAND+UP+SHOUT+OUT) | Shade Township, PA 15926, USA | United States | North America |
| 28 | TheraTalk | **✓** |  |  | [TheraTalk](https://play.google.com/store/apps/developer?id=TheraTalk) | Hong Kong, China | China | East Asia |
| 29 | TrackActive Me: Virtual Physio | **✓** |  |  | [Active Health Tech Ltd](https://play.google.com/store/apps/developer?id=Active+Health+Tech+Ltd) | 55 Mark Lane London United Kingdom EC3R 7NE | United Kingdom | Northwestern Europe |
| 30 | Virtual Assistant: AI Friend |  |  |  | [end-plif!](https://play.google.com/store/apps/dev?id=8036064890445184708) | [Rua Leônidas Pimentel, Centro, BARREIRINHA, AM](https://maps.google.com/?q=Rua%20Le%C3%B4nidas%20Pimentel,%20Centro,%20BARREIRINHA,%20AM) | Brazil | South America |
| 31 | WellWisher |  |  |  | [Hari Jyothula](https://play.google.com/store/apps/developer?id=Hari+Jyothula) | Department of Information Technology, Vignan's Institute of Engineering for Women, Visakhapatnam | India | South Asia |
| 32 | WingMan For Addiction | **✓** |  |  | [Addiction Resource Systems, Inc.](https://play.google.com/store/apps/developer?id=Addiction+Resource+Systems,+Inc.) | 2780 NE 183rd St, Suite 2117 Aventura, FL 33160 | United States | North America |
| 33 | Woebot: Your Self-Care Expert | **✓** |  |  | [Woebot Health Inc](https://play.google.com/store/apps/developer?id=Woebot+Health+Inc) | 1460 Mission Street San Francisco, CA 94103 | United States | North America |
| 34 | Wysa: Mental Health Support | **✓** |  |  | [Touchkin](https://play.google.com/store/apps/dev?id=6520130637621273347) | Touchkin Eservices Private Limited, No. 532, "Manjusha", First Floor, 2nd main, 16th Cross, II stage, Indiranagar, Bengaluru 560038 | India | South Asia |
| 35 | Ziver - Always On | **✓** |  |  | [Digital Industria Ltd](https://play.google.com/store/apps/developer?id=Digital+Industria+Ltd) | Unit 11, Hove Business Centre Fonthill Road Hove East Sussex BN3 6HA | United Kingdom | Northwestern Europe |
| 36 | 7 Cups: Therapy & Support |  | **✓** |  | [7 Cups](https://play.google.com/store/apps/developer?id=7+Cups+of+Tea)  [of Tea](https://play.google.com/store/apps/developer?id=7+Cups+of+Tea) | 3714 Jefferson Boulevard, Suite B Virginia Beach, VA 23455 | United States | North America |

*Note:* Please note that the provided list is organized in alphabetical order. Among the applications, three are accessible on both iOS and Android platforms. However, it’s important to note that the names of these applications may differ based on the respective app stores. The names listed on the left side correspond to their designations in the iOS store, while those on the right-hand side correspond to their nomenclature in the Android store. For instance, in the iOS store, the application is denoted as "Amaha: Mental Health Self-Care," while in the Android store, it goes by the name "Amaha: Anxiety Sleep Self-Care."
