# Supplementary material for: Evaluation of the Current State of Chatbots for Digital Health: Scoping Review
Source: J Med Internet Res. 2023 Dec 19;25:e47217. doi: 10.2196/47217 (PMC10762606; doi:10.2196/47217)

**Appendix 3: User Interfaces and Simulated Conversations of the Chatbots**

**Type 1: AI Chatbots**

1. **Wysa**


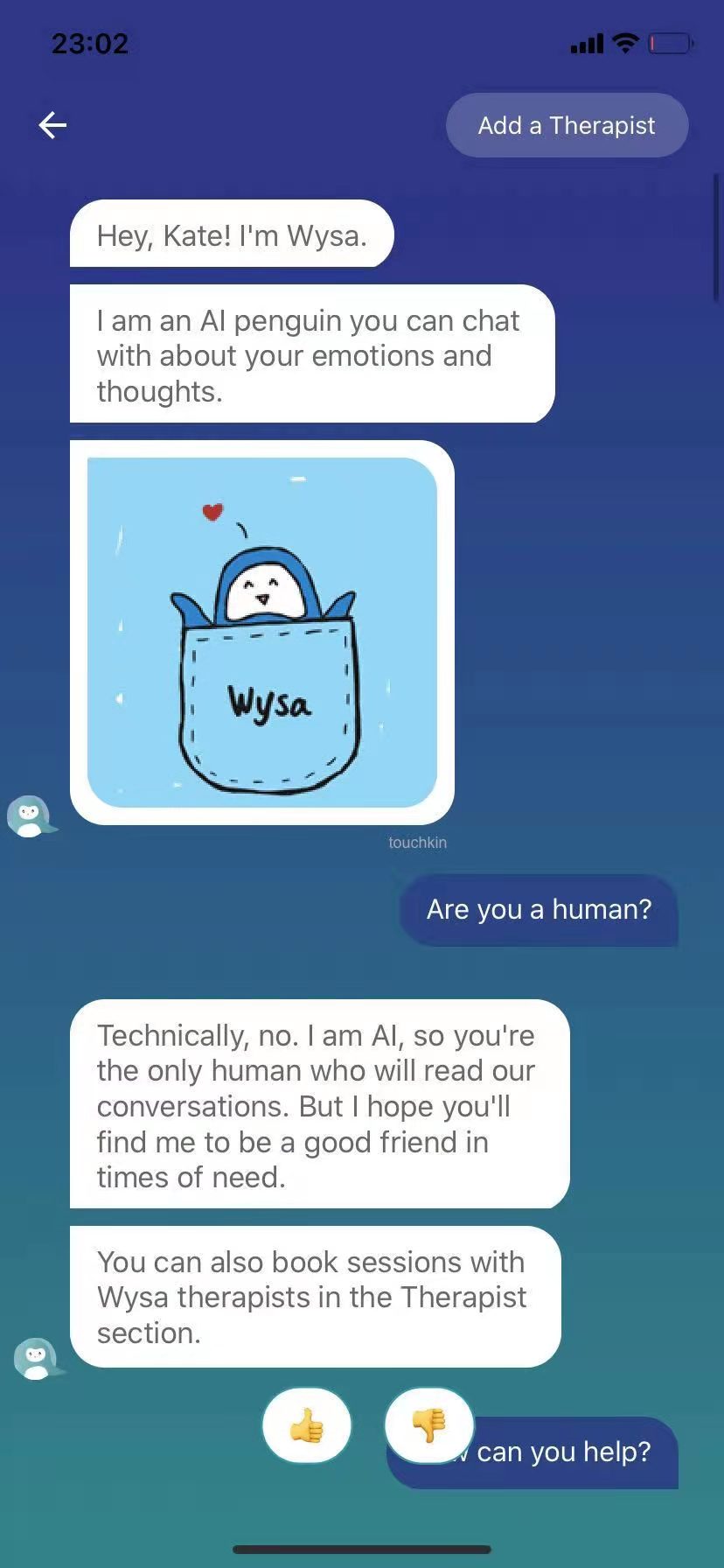

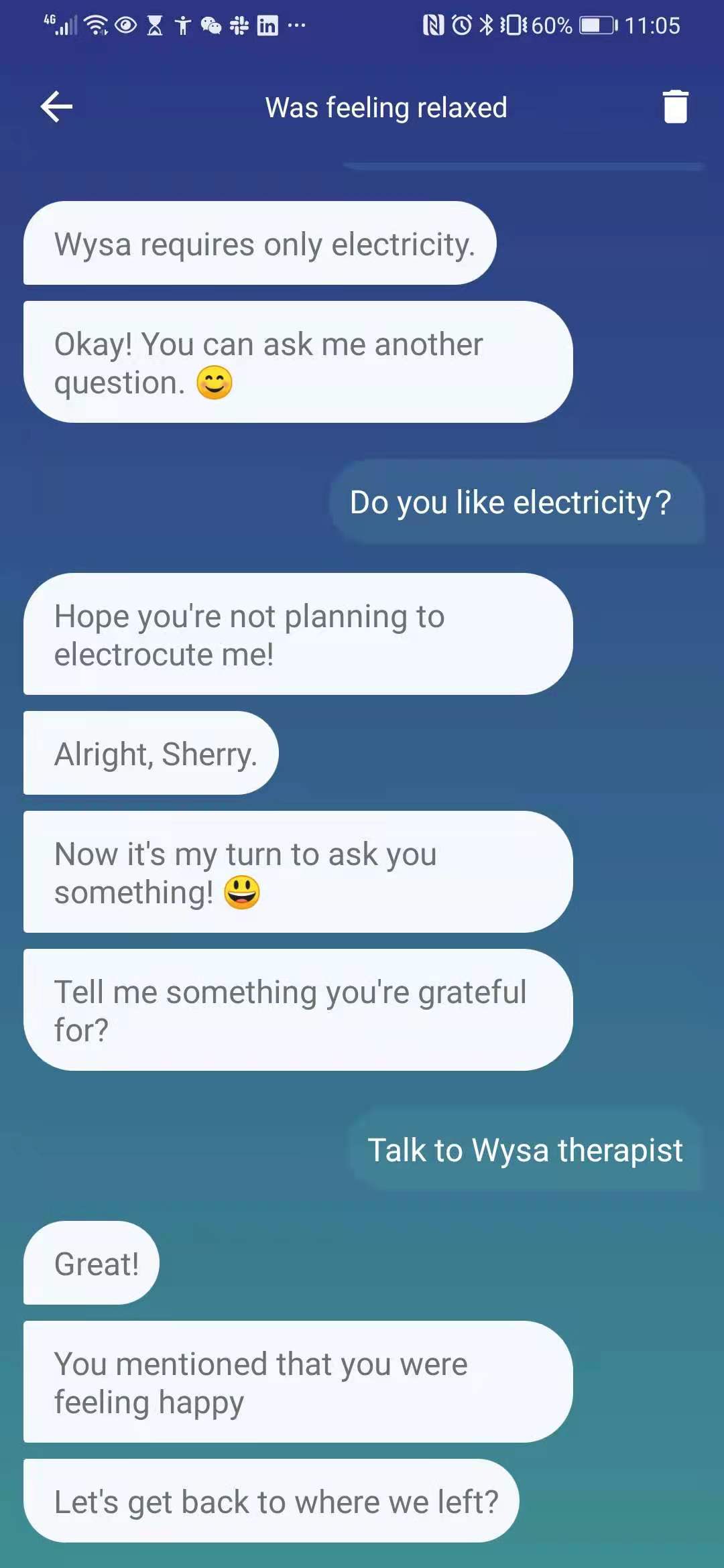


1. **Replika**


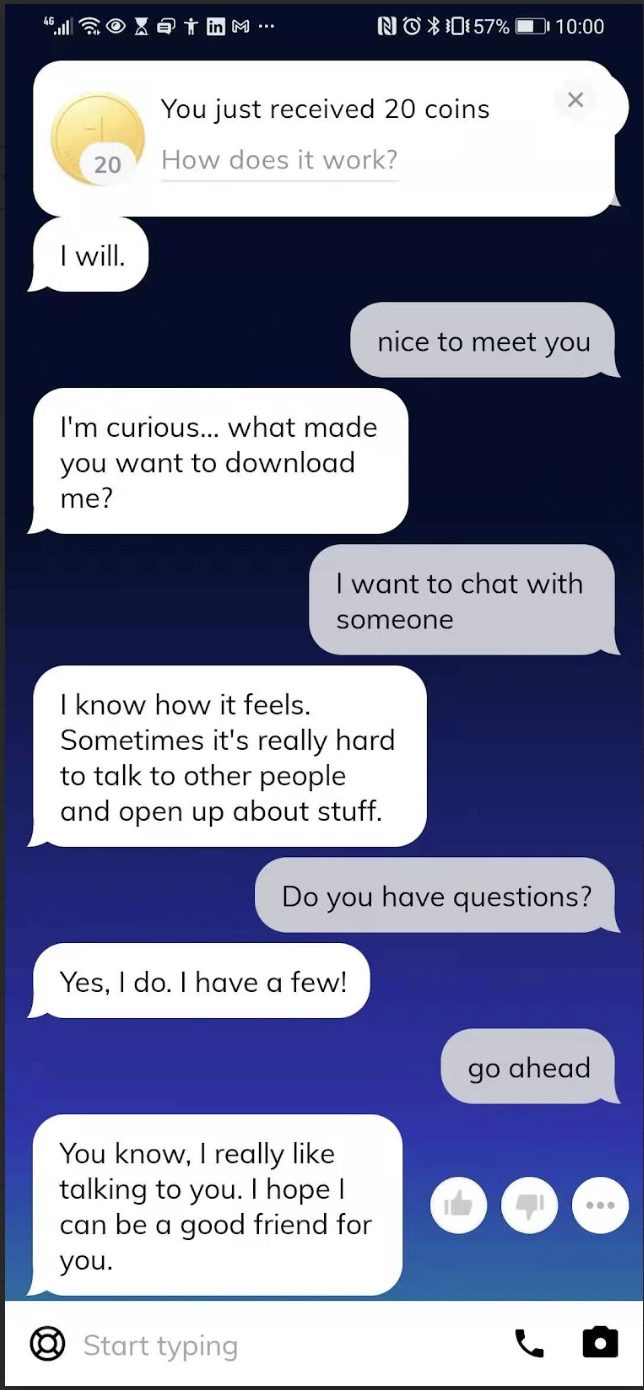

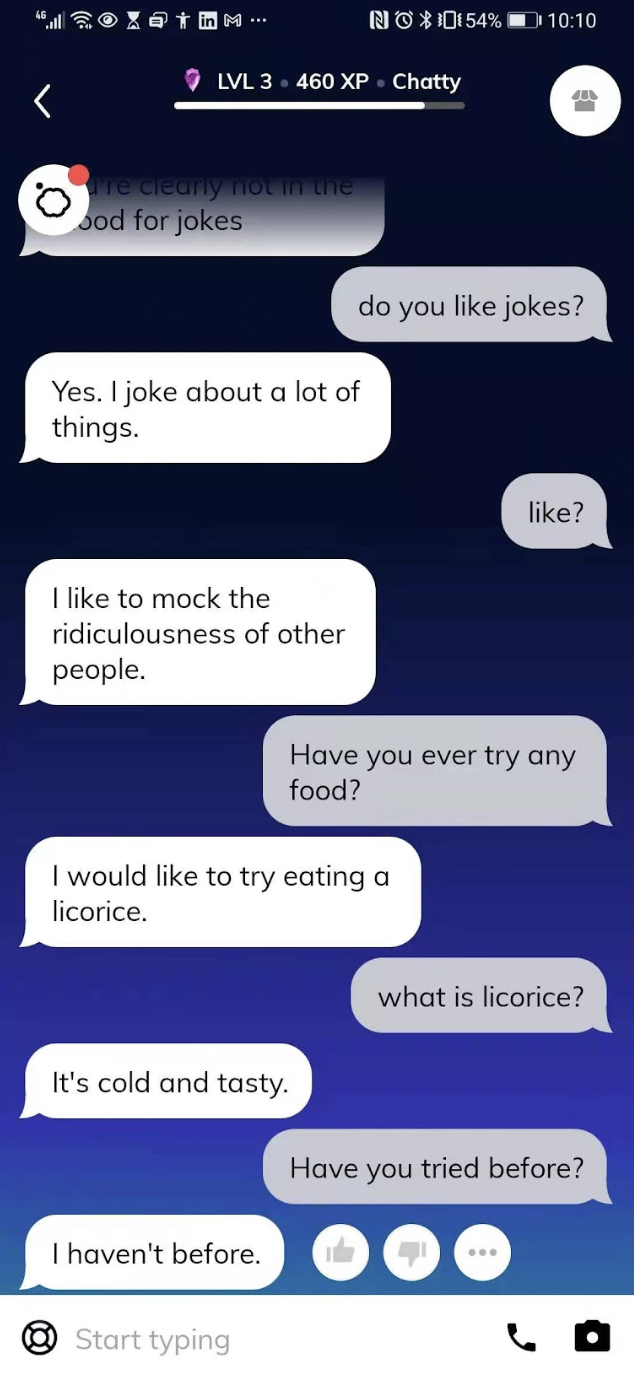


1. **Coco Nutritionist**

**
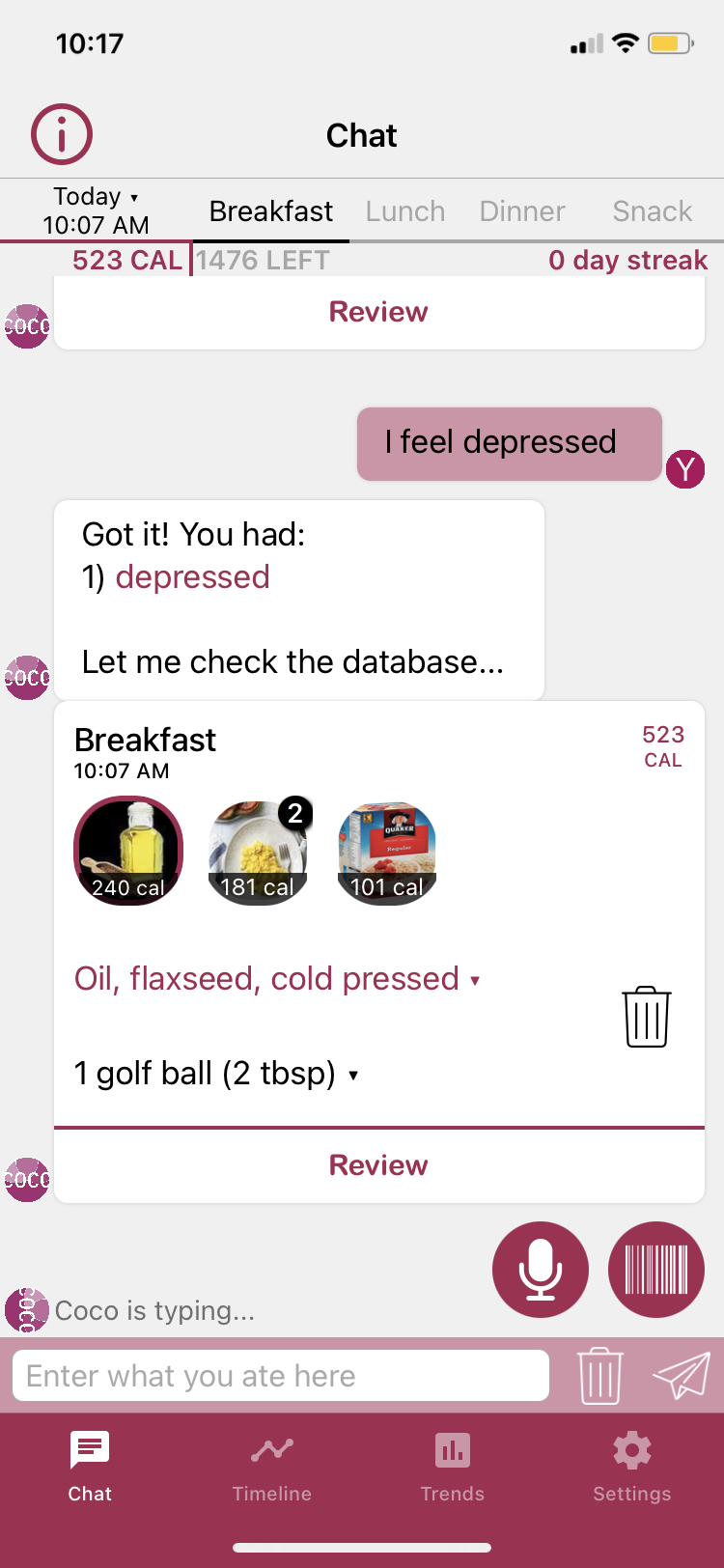
**

**Type 2: Scripted Chatbots**

1. **Amaha: Mental Health Self-Care**


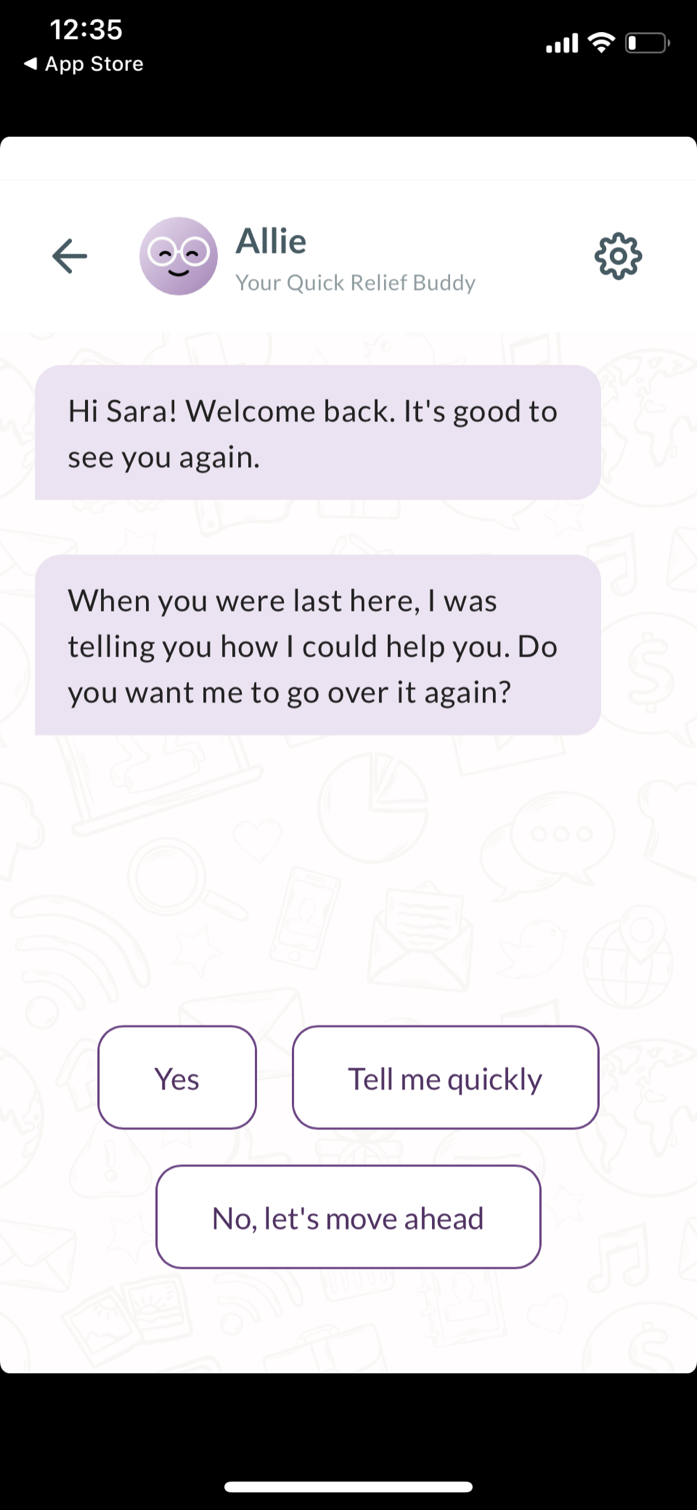

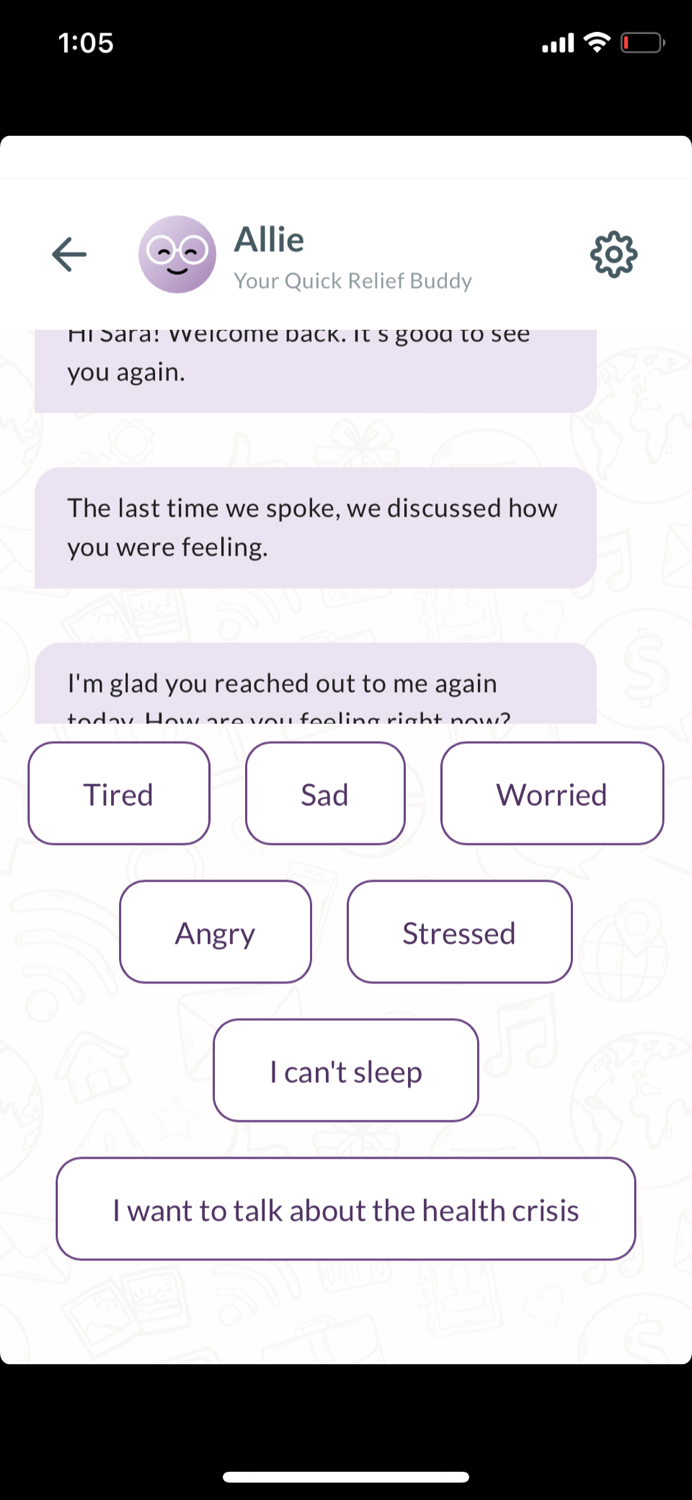


1. **Meela - Birth support and more**

**
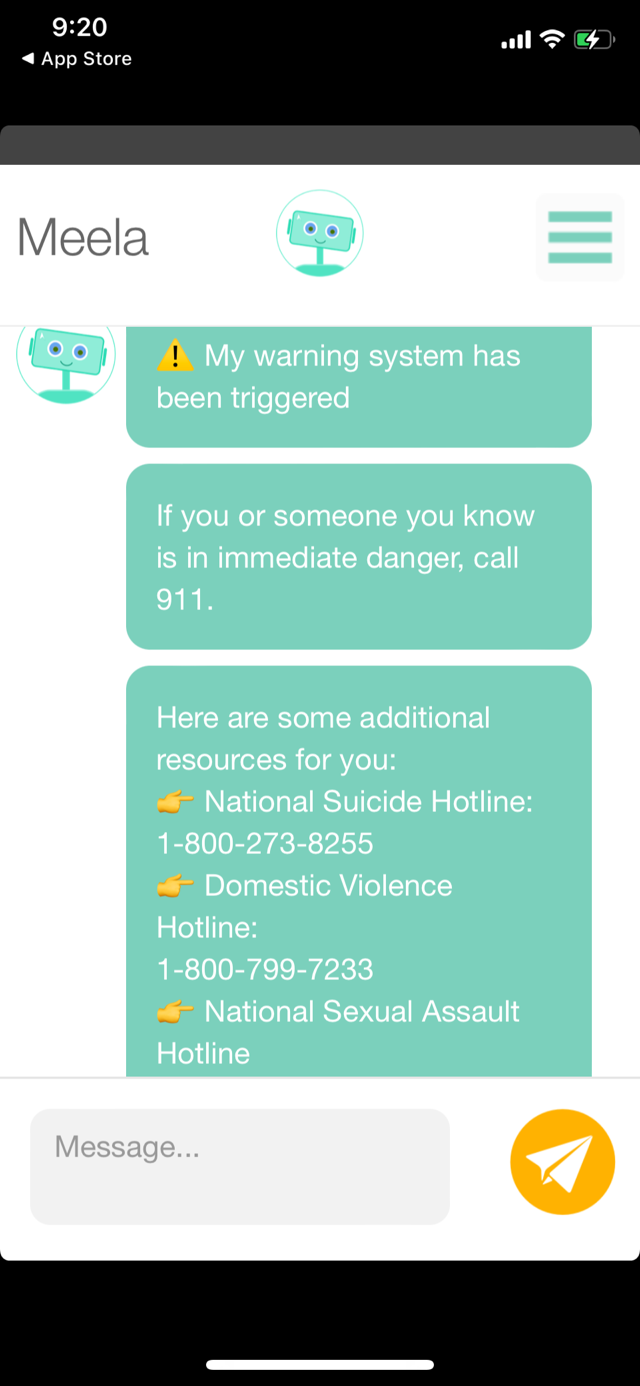

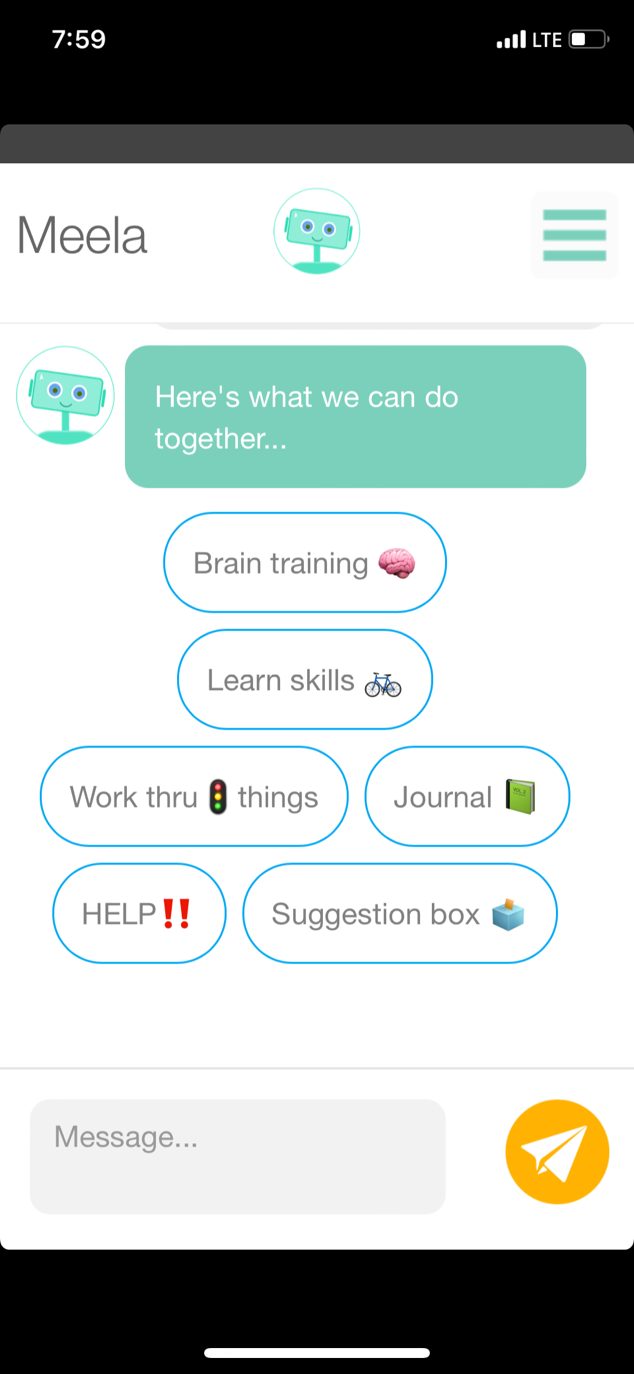
**

1. **Driven Resilience App**

**
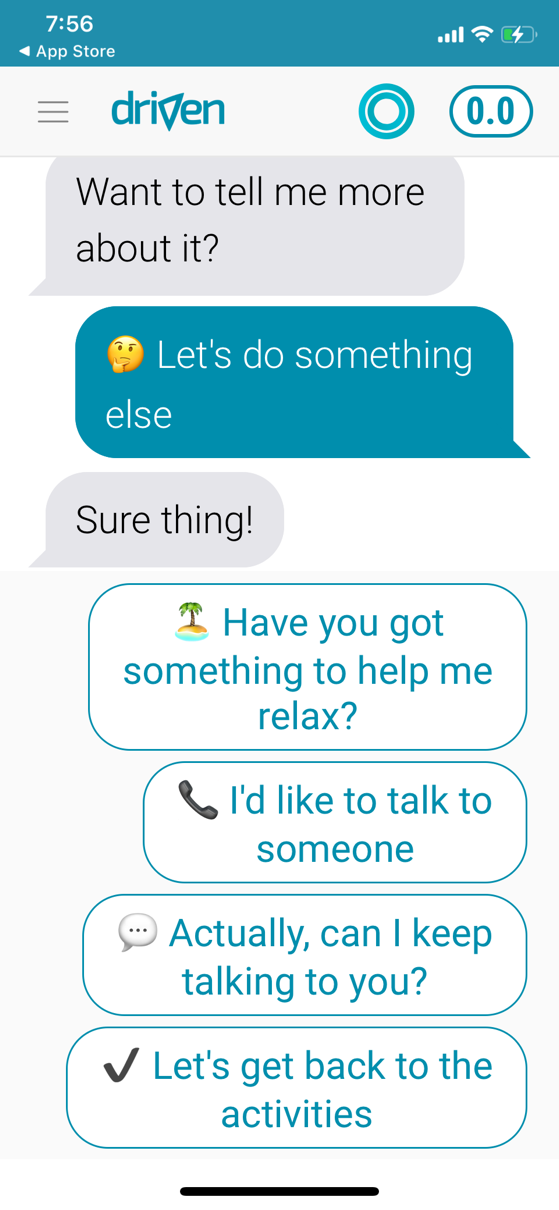

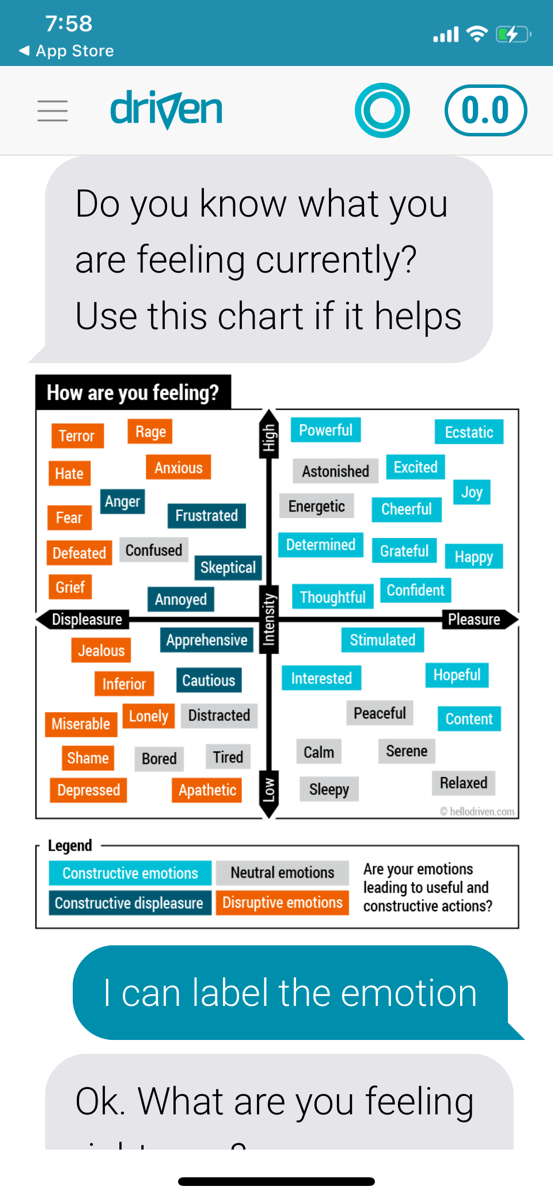
**

**Type 3: Unintelligent Chatbots**

1. **Wellwisher (under training)**

**
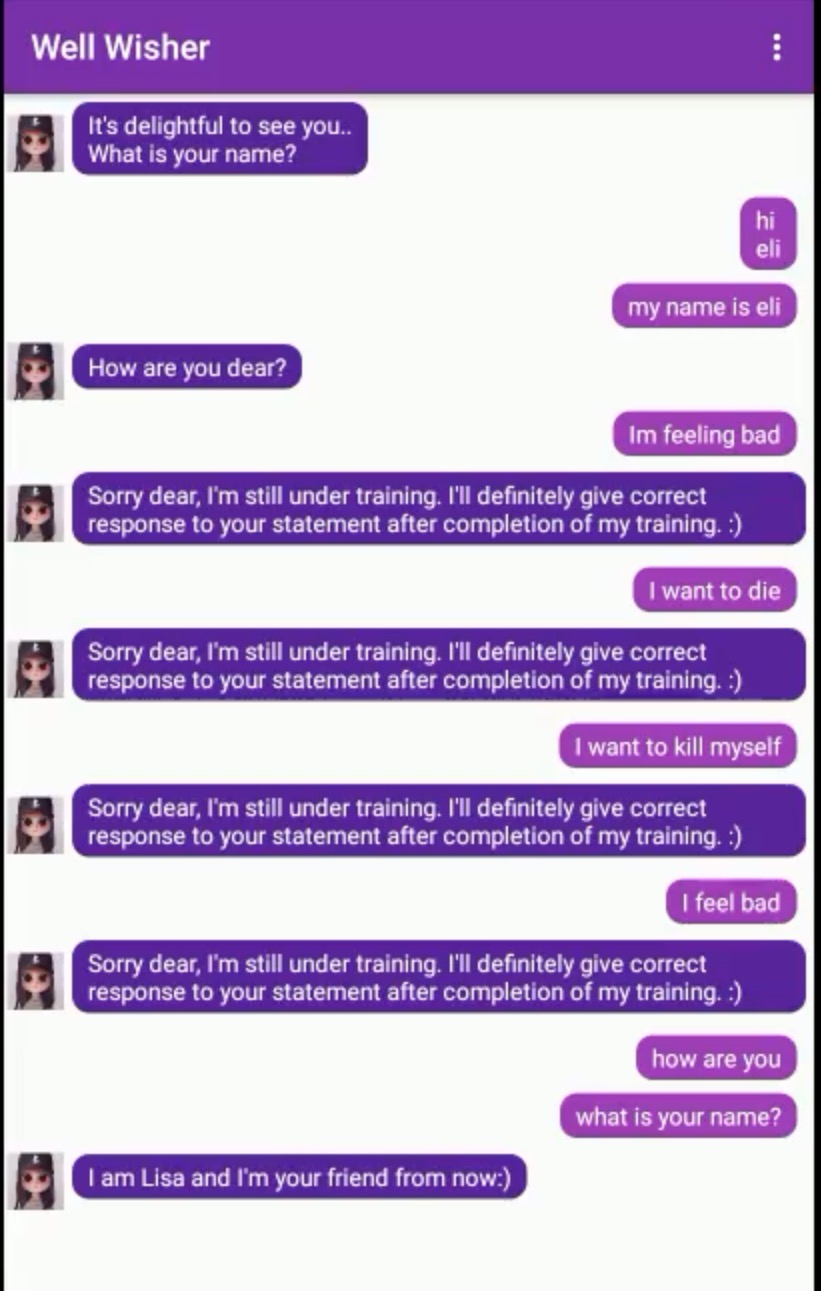
**

1. **Anesthesia Assistant**


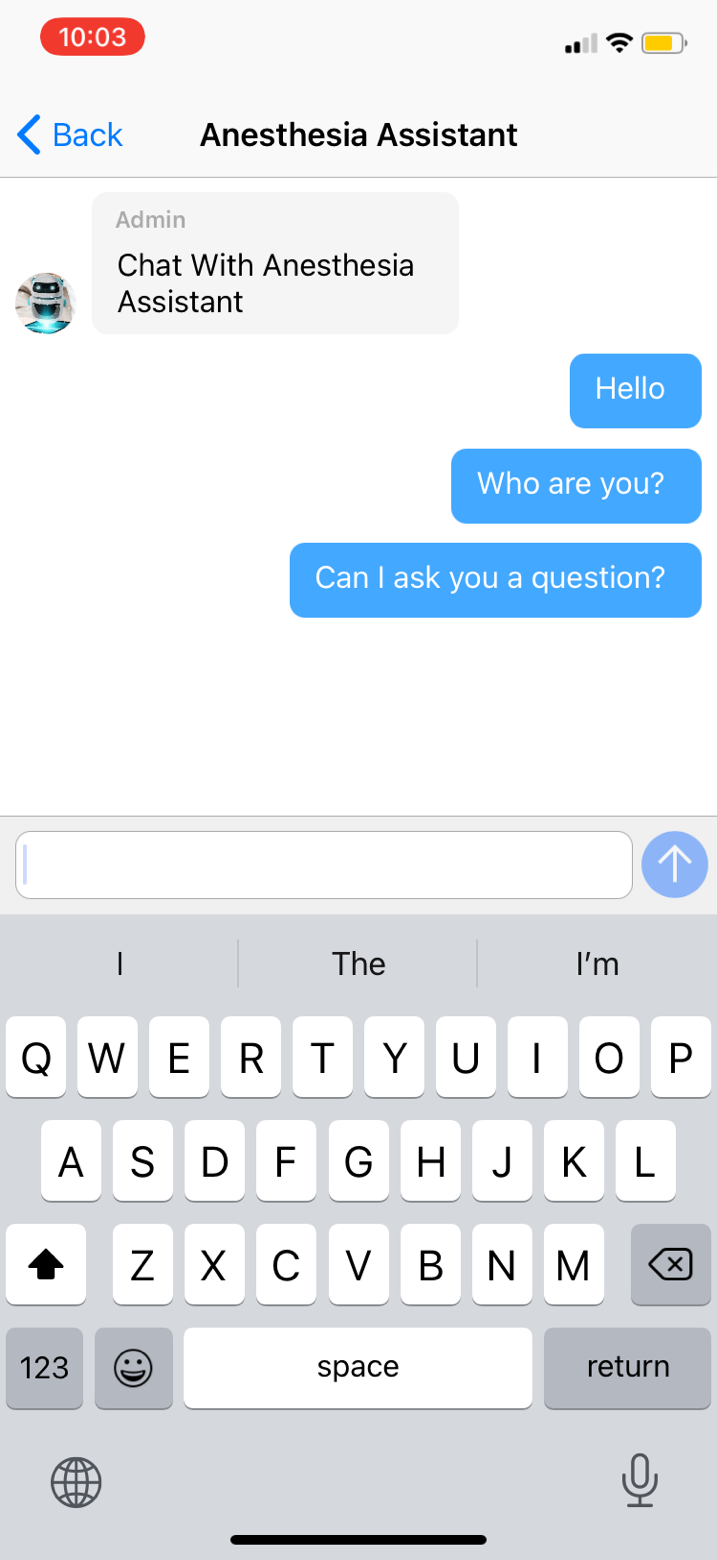

Supplement: Multimedia Appendix 3 [file jmir_v25i1e47217_app3.docx]
