# Supplementary material for: Evaluation of the Current State of Chatbots for Digital Health: Scoping Review
Source: J Med Internet Res. 2023 Dec 19;25:e47217. doi: 10.2196/47217 (PMC10762606; doi:10.2196/47217)
Supplement: Multimedia Appendix 4 [file jmir_v25i1e47217_app4.docx]

**Table S1. Assessment scheme/tool informed by the framework developed by the team**

| **Themes** | **Categories** | **Coding question** |
| --- | --- | --- |
| **Chatbot characteristics** | Target age group | What is the age group that the chatbots are targeted towards? |
|  | Developer | Who developed the app? |
|  | Size | What is the size of the chatbot? |
|  | Category | Under which category is the chatbot listed in the store? |
|  | Anthropomorphic cues | What are the anthropomorphic cues related to identity, such as name, gender, and others? |
| **User backgrounds** | Demographic characteristics collected | What demographic characteristics are collected by the chatbot, such as name, gender, age, and others? |
| **Communication models** | Media presented in the chatbot | What types of media are presented in the chatbot, such as text, speech, image, gif, animation, video, emoji, and others? |
|  | Modalities available by the users | What modalities are available for users to interact with the chatbot, such as text, speech, image, gif, animation, video, emoji, and others |
|  | Scripted chatbots | Does the chatbot solely offer predetermined responses and lacks the ability to adjust its responses based on the user's specific context and needs? |
|  | AI chatbots | Is the chatbot capable of providing responses that are personalized to the user's specific needs and context? |
| **Building relational capacity** | Social dialogue | Is the chatbot capable of engaging in social dialogue? |
|  | Conversation initiation | If yes to “social dialogue,” who initiated the conversation? |
|  | Empathy | Is the chatbot capable of exhibiting empathy, such as demonstrating understanding and emotional support? |
|  | Humor | Does the chatbot possess a sense of humor or the ability to express jokes or amusing responses? |
|  | Self-disclosure | Does the chatbot can self-disclose, that is, reveal personal opinions, thoughts or feelings? |
|  | Meta-relational communication | Can the chatbot acknowledge and discuss the relationship with the user and periodically check in to ensure a smooth interaction? |
| **Personalization** | Chat history tracking | Does the chatbot maintain a record of chat history, that is, keep a log of past conversations with the user? |
|  | Persistent memory | Does the chatbot possess persistent memory, such as the ability to recall and remember the user's preferences and behaviors over time? |
|  | Constructing user models | How does the chatbot construct the user models for personalization? Is it through the automatic analysis of observed user activities and interactions (implicit) or by explicitly collecting information through user input and participation (explicit)? |
|  | Chatbot features | The chatbot's personalization options may include customizing content, modifying the user interface to change how information is presented, selecting a preferred delivery channel (such as text or voice messages), and choosing between free or paid versions that offer different levels of functionality. |
| **Interaction** | Purpose of the chatbot | What is the primary purpose of the chatbot, such as providing therapy or counseling, professional skills training, screening for asymptomatic individuals, self-management or monitoring, educational health information, or diagnostic testing? |
|  | Targeted users | Which group of users is the chatbot primarily designed for, such as the general public, students, or therapists and physicians? |
| **Response to suicidal thoughts** | Responding to suicidal thoughts | Can the chatbot effectively address and respond to suicidal thoughts, offering emergency resources, providing access to direct helplines, implementing safety check functions, and taking other proactive measures beyond simply exhibiting empathy? |
|  | Responses | In the context of suicidal thoughts, what strategies or resources does the chatbot employ to respond effectively and appropriately? |
| **In-app user experience** | Ratings | What is the rating of the chatbot in the app store or marketplace? |
|  | User feedback | The user experience of the chatbots, including convenience, satisfaction, and usefulness, will be evaluated to assess the mechanisms and outcomes associated with their use. |

**Table S2 Descriptive statistics for the features of the coded chatbots (n=36)**

| **Categories** | **Subcategories** | **n (%)** | **Chatbot lists** |
| --- | --- | --- | --- |
| **Chatbot characteristics** | | | |
| Target age group | Everyone, 4+ | 17 (47.2) | AskFirst (formerly Ask NHS), Chat Bot, ChatPal, Elena-The Medical Assistant, Laura,  Naturopathy Assistant, SophieBot AI, WellWisher, Ziver - Always On, IWill Care, Driven Resilience App, PocketFriend Digital Therapy, Reflectly - Journal & AI Diary, Coco Nutritionist, Elysai: Talk to AI Friends, Virtual Assistant: AI Friend, Sensely |
|  | Teens | 10 (27.8) | Magnify Wellness, TheraTalk, Woebot: Your Self-Care Expert, Meela - Birth support and more, 7 Cups: Therapy & Support, TrackActive Me: Virtual Physio, MARCo: Mental Health Friend, NatHealth VA, RiseUp: AI based CBT Therapy, Wysa: Mental Health Support |
|  | Mature 17+ | 8  (22.2) | Amaha: Mental Health Self-Care, WingMan For Addiction, Anesthesia Assistant, Mediktor - Symptom Checker, MayaMD, OWL Cancer Survivor Platform, SophieBot AI, Replika: My AI Friend |
| Developer | Individuals | 6  (16.2) | PocketFriend Digital Therapy, Naturopathy Assistant, Coco Nutritionist, RiseUp: AI based CBT Therapy, WellWisher, Magnify Wellness |
| Size | Less than 10M | 6  (16.7) | Elena-The Medical Assistant, ChatPal, Chat Bot, Naturopathy Assistant, SophieBot AI, WellWisher |
| Category | Health & Fitness | 22 (61.1) | Buoy Health, Chat Bot, ChatPal, Magnify Wellness, Naturopathy Assistant, Replika: My AI Friend, TheraTalk, WellWisher, Meela - Birth support and more, Amaha: Mental Health Self-Care, 7 Cups: Therapy & Support, Driven Resilience App, PocketFriend Digital Therapy, Reflectly - Journal & AI Diary, Coco Nutritionist, Elysai: Talk to AI Friends, MARCo: Mental Health Friend, OWL Cancer Survivor Platform, RiseUp: AI based CBT Therapy, Sensely, SophieBot AI, Wysa: Mental Health Support |
|  | Medical | 9  (25) | AskFirst (formerly Ask NHS), SophieBot AI, Woebot: Your Self-Care Expert, IWill Care, MayaMD, Mediktor - Symptom Checker, TrackActive Me: Virtual Physio, Anesthesia Assistant, NatHealth VA |
|  | Entertainment | 1 (2.8) | Laura |
|  | Lifestyle & Social Networking | 3 (8.3) | Ziver - Always On, Virtual Assistant: AI Friend, WingMan For Addiction |
| Anthropomorphic cues | Name identity | 24  (66.7) | Elena-The Medical Assistant, Wysa: Mental Health Support, Amaha: Mental Health Self-Care, IWill Care, Woebot: Your Self-Care Expert, Meela - Birth support and more, ChatPal, Chat Bot, Ziver - Always On, Coco Nutritionist, OWL Cancer Survivor Platform, RiseUp: AI based CBT Therapy, SophieBot AI, Reflectly - Journal & AI Diary, ChatGPT, TheraTalk, WellWisher, MARCo: Mental Health Friend, Laura, AskFirst (formerly Ask NHS), Replika: My AI Friend, Elysai: Talk to AI Friends, Magnify Wellness, 7 Cups: Therapy & Support |
|  | Chatbots identity | 21  (58.3) | Elena-The Medical Assistant, Wysa: Mental Health Support, Amaha: Mental Health Self-Care, IWill Care, Woebot: Your Self-Care Expert, ChatPal, Chat Bot, Naturopathy assistant AI chatbot, Ziver - Always On, RiseUp: AI based CBT Therapy, Virtual Assistant: AI Friend, SophieBot AI, Reflectly - Journal & AI Diary, ChatGPT, WellWisher,  MARCo: Mental Health Friend, Laura, Replika: My AI Friend, Elysai: Talk to AI Friends, Magnify Wellness, 7 Cups: Therapy & Support |
|  | Gender identity | 7  (19.4) | Chat Bot, ChatGPT, WellWisher, MARCo: Mental Health Friend, Laura, Replika: My AI Friend, Elysai: Talk to AI Friends |
|  | No anthropomorphic | 1  (2.8) | Buoy Health |
| **User Backgrounds** | | | |
| Demographic characteristics collected | User’s name | 23  (63.9) | Elena-The Medical Assistant, Wysa: Mental Health Support, Amaha: Mental Health Self-Care, Woebot: Your Self-Care Expert, Meela - Birth support and more, PocketFriend Digital Therapy, ChatPal, Buoy Health, Coco Nutritionist, MayaMD, OWL Cancer Survivor Platform, RiseUp: AI based CBT Therapy, Virtual Assistant: AI Friend, Sensely, SophieBot AI, Reflectly - Journal & AI Diary, TheraTalk, WellWisher, MARCo: Mental Health Friend, Laura, AskFirst (formerly Ask NHS), Elysai: Talk to AI Friends, 7 Cups: Therapy & Support |
|  | User’s gender | 9 (25) | Elena-The Medical Assistant, IWill Care, ChatPal, Buoy Health, MayaMD, Sensely,  SophieBot AI, Magnify Wellness, AskFirst (formerly Ask NHS) |
|  | User’s age | 8 (22.2) | IWill Care, ChatPal, Buoy Health, Mediktor - Symptom Checker, Sensely, Magnify Wellness, Laura, AskFirst (formerly Ask NHS) |
|  | User’s location | 3 (8.3) | ChatPal, Buoy Health, Mediktor - Symptom Checker |
|  | User’s occupation | 1 (2.8) | Replika: My AI Friend |
|  | No user backgrounds | 10 (27.8) | Chat Bot, Naturopathy Assistant,  SophieBot AI, Ziver - Always On, Magnify Wellness, Driven Resilience App, TrackActive Me: Virtual Physio, Anesthesia Assistant,  NatHealth VA, WingMan For Addiction |
| **Communication Models** | | | |
| Media presented in the chatbot | Animations | 9 (25) | Elena-The Medical Assistant, Wysa: Mental Health Support, Sensely, SophieBot AI, MARCo: Mental Health Friend, Laura, AskFirst (formerly Ask NHS), Replika: My AI Friend, Elysai: Talk to AI Friends |
|  | Speech/audio | 11  (30.6) | Elena-The Medical Assistant, Wysa: Mental Health Support, Amaha: Mental Health Self-Care, WingMan For Addiction, NatHealth VA,  Virtual Assistant: AI Friend, SophieBot AI, WellWisher, MARCo: Mental Health Friend, AskFirst (formerly Ask NHS), Elysai: Talk to AI Friends |
|  | Static images | 11 (30.6) | Wysa: Mental Health Support, Driven Resilience App, TrackActive Me: Virtual Physio, Mediktor - Symptom Checker, Coco Nutritionist, NatHealth VA, OWL Cancer Survivor Platform, Reflectly - Journal & AI Diary, MARCo: Mental Health Friend, Laura, Magnify Wellness |
|  | Emojis | 11 (30.6) | Wysa: Mental Health Support, Amaha: Mental Health Self-Care, Woebot: Your Self-Care Expert, Meela - Birth support and more, Driven Resilience App, ChatPal, NatHealth VA, RiseUp: AI based CBT Therapy, ChatGPT, Replika: My AI Friend, Elysai: Talk to AI Friends |
|  | Video | 2 (5.6) | Wysa: Mental Health Support, Driven Resilience App |
| Modalities available by the users | Emoji | 14 (38.9) | Elena-The Medical Assistant, Replika: My AI Friend, SophieBot AI, Woebot: Your Self-Care Expert, Ziver - Always On, ChatGPT, Coco Nutritionist, MARCo: Mental Health Friend, NatHealth VA, OWL Cancer Survivor Platform, RiseUp: AI based CBT Therapy, WingMan For Addiction, Wysa: Mental Health Support, 7 Cups: Therapy & Support |
|  | Direct speech | 11 (30.6) | AskFirst (formerly Ask NHS), Chat Bot, WellWisher, Coco Nutritionist,  Elysai: Talk to AI Friends, MARCo: Mental Health Friend, NatHealth VA, OWL Cancer Survivor Platform, Virtual Assistant: AI Friend, Sensely, WingMan For Addiction |
|  | Images | 3 (8.3) | Replika: My AI Friend, Coco Nutritionist, NatHealth VA |
| Scripted chatbots | | 19 (52.8) | Amaha: Mental Health Self-Care, IWill Care, Meela - Birth support and more, Driven Resilience App, TrackActive Me: Virtual Physio, PocketFriend Digital Therapy, ChatPal, Buoy Health, Mediktor - Symptom Checker, MayaMD, NatHealth VA, RiseUp: AI based CBT Therapy, Virtual Assistant: AI Friend, Sensely, TheraTalk, MARCo: Mental Health Friend, AskFirst (formerly Ask NHS), Magnify Wellness, 7 Cups: Therapy & Support |
| AI chatbots | | 15 (41.7) | Elena-The Medical Assistant, Wysa: Mental Health Support, WingMan For Addiction, Woebot: Your Self-Care Expert, Chat Bot, Naturopathy Assistant, Ziver - Always On, Coco Nutritionist, OWL Cancer Survivor Platform, SophieBot AI, Reflectly - Journal & AI Diary, ChatGPT, Laura, Replika: My AI Friend, Elysai: Talk to AI Friends |
| Unintelligent chatbots^[[1]](#footnote-1)^ | | 2 (5.6) | Anesthesia Assistant, WellWisher |
| **Building Relational Capacity** | | | |
| Social dialogue and initiation conversation | Initiate social dialogues | 22 (61.1) | Wysa: Mental Health Support, Amaha: Mental Health Self-Care, IWill Care, Woebot: Your Self-Care Expert, Meela - Birth support and more, PocketFriend Digital Therapy, ChatPal, Chat Bot, Mediktor - Symptom Checker, OWL Cancer Survivor Platform, RiseUp: AI based CBT Therapy, Sensely, Reflectly - Journal & AI Diary, ChatGPT, TheraTalk, WellWisher, MARCo: Mental Health Friend, Laura, AskFirst (formerly Ask NHS), Replika: My AI Friend, Elysai: Talk to AI Friends, 7 Cups: Therapy & Support |
|  | Responding to social dialogues | 10 (27.8) | Elena-The Medical Assistant, WingMan For Addiction, Meela - Birth support and more, ChatPal, Chat Bot, Naturopathy Assistant, Ziver - Always On, ChatGPT, SophieBot AI, Replika: My AI Friend |
| Empathy | Exhibiting empathy | 23 (63.9) | AskFirst (formerly Ask NHS), ChatPal, Elena-The Medical Assistant, Laura, 7 Cups: Therapy & Support, Replika: My AI Friend, WellWisher, Woebot: Your Self-Care Expert, Ziver - Always On, Meela - Birth support and more, Amaha: Mental Health Self-Care, IWill Care, Magnify Wellness, Driven Resilience App, PocketFriend Digital Therapy, ChatGPT, Elysai: Talk to AI Friends, MARCo: Mental Health Friend, OWL Cancer Survivor Platform, RiseUp: AI based CBT Therapy, Sensely, WingMan For Addiction, Wysa: Mental Health Support |
| Humor | Human function | 9 (25) | Elena-The Medical Assistant, Wysa: Mental Health Support, WingMan For Addiction, NatHealth VA, ChatGPT, MARCo: Mental Health Friend, Laura, Replika: My AI Friend, Magnify Wellness |
| Self-disclosure | | 21 (58.3) | Amaha: Mental Health Self-Care, IWill Care, Meela - Birth support and more, Driven Resilience App, TrackActive Me: Virtual Physio, Anesthesia Assistant, PocketFriend Digital Therapy, Buoy Health, Chat Bot, Mediktor - Symptom Checker, Ziver - Always On, Coco Nutritionist, MayaMD, OWL Cancer Survivor Platform, Sensely, Reflectly - Journal & AI Diary, SophieBot AI, TheraTalk, MARCo: Mental Health Friend, Magnify Wellness, 7 Cups: Therapy & Support |
| Meta-relational communication | | 18 (50) | Elena-The Medical Assistant, Wysa: Mental Health Support, Amaha: Mental Health Self-Care, IWill Care, Woebot: Your Self-Care Expert, Meela - Birth support and more, Driven Resilience App, ChatPal, RiseUp: AI based CBT Therapy, SophieBot AI, ChatGPT, WellWisher, Laura, AskFirst (formerly Ask NHS), Replika: My AI Friend, Elysai: Talk to AI Friends, Magnify Wellness, 7 Cups: Therapy & Support |
| **Personalization** | | | |
| Chat history tracking | | 17 (47.2) | Wysa: Mental Health Support, WingMan For Addiction, Woebot: Your Self-Care Expert, Meela - Birth support and more, ChatPal, RiseUp: AI based CBT Therapy, Virtual Assistant: AI Friend, Sensely, SophieBot AI, Reflectly - Journal & AI Diary, ChatGPT, TheraTalk, WellWisher, MARCo: Mental Health Friend, Replika: My AI Friend, Elysai: Talk to AI Friends, 7 Cups: Therapy & Support |
| Persistent memory | | 10 (27.8) | Wysa: Mental Health Support, Amaha: Mental Health Self-Care, Woebot: Your Self-Care Expert, PocketFriend Digital Therapy, ChatPal, Reflectly - Journal & AI Diary, Laura, AskFirst (formerly Ask NHS), Replika: My AI Friend, 7 Cups: Therapy & Support |
| Constructing user models | Explicit | 15 (41.7) | Elena-The Medical Assistant, Wysa: Mental Health Support, Amaha: Mental Health Self-Care, WingMan For Addiction, TrackActive Me: Virtual Physio, Buoy Health, RiseUp: AI based CBT Therapy, Reflectly - Journal & AI Diary, TheraTalk, MARCo: Mental Health Friend, Laura, AskFirst (formerly Ask NHS), Replika: My AI Friend, Elysai: Talk to AI Friends, 7 Cups: Therapy & Support |
|  | Implicit | 10 (27.8) | Wysa: Mental Health Support, WingMan For Addiction, Woebot: Your Self-Care Expert, PocketFriend Digital Therapy, RiseUp: AI based CBT Therapy, Sensely, Reflectly - Journal & AI Diary, MARCo: Mental Health Friend, Elysai: Talk to AI Friends, Magnify Wellness |
| Chatbot features | Content | 22 (61.1) | Elena-The Medical Assistant, Wysa: Mental Health Support, Amaha: Mental Health Self-Care, IWill Care, WingMan For Addiction, Woebot: Your Self-Care Expert, Meela - Birth support and more, PocketFriend Digital Therapy, Buoy Health, RiseUp: AI based CBT Therapy, Sensely, Reflectly - Journal & AI Diary, ChatGPT, SophieBot AI, TheraTalk, MARCo: Mental Health Friend, Laura, AskFirst (formerly Ask NHS), Replika: My AI Friend, Elysai: Talk to AI Friends, Magnify Wellness, 7 Cups: Therapy & Support |
|  | User interface | 5 (13.9) | Wysa: Mental Health Support, Amaha: Mental Health Self-Care, ChatGPT, MARCo: Mental Health Friend, AskFirst (formerly Ask NHS) |
|  | Delivery channel | 1 (2.8) | WingMan For Addiction |
|  | Functionality | 6 (16.7) | Wysa: Mental Health Support, TrackActive Me: Virtual Physio, OWL Cancer Survivor Platform, ChatGPT, Replika: My AI Friend, TheraTalk |
|  | Enchanced functionality behind paywalls | 6 (16.7) | Wysa: Mental Health Support, TrackActive Me: Virtual Physio, OWL Cancer Survivor Platform, ChatGPT, Replika: My AI Friend, TheraTalk |
| **Interaction** | | | |
| Purpose of the chatbot | Therapy/counseling | 18 (50.0) | Elena-The Medical Assistant, Wysa: Mental Health Support, Amaha: Mental Health Self-Care, IWill Care, WingMan For Addiction, Woebot: Your Self-Care Expert, Meela - Birth support and more, Driven Resilience App, PocketFriend Digital Therapy, ChatPal, Buoy Health, NatHealth VA, RiseUp: AI based CBT Therapy, ChatGPT, TheraTalk,  MARCo: Mental Health Friend, Replika: My AI Friend, Elysai: Talk to AI Friends |
|  | Educational | 5 (13.9) | Elena-The Medical Assistant, Naturopathy Assistant, OWL Cancer Survivor Platform, SophieBot AI, SophieBot AI |
|  | Diagnosis | 4 (11.1) | NatHealth VA, Sensely, ChatGPT, Wysa: Mental Health Support |
|  | Screening | 4 (11.1) | Buoy Health, Chat Bot, RiseUp: AI based CBT Therapy, AskFirst (formerly Ask NHS) |
|  | Self-management | 5 (13.9) | ChatPal, RiseUp: AI based CBT Therapy, Virtual Assistant: AI Friend, Reflectly - Journal & AI Diary, AskFirst (formerly Ask NHS) |
| Targeted users | General public | 32 (88.9) | Elena-The Medical Assistant, Amaha: Mental Health Self-Care, IWill Care, WingMan For Addiction, Woebot: Your Self-Care Expert, Meela - Birth support and more, Driven Resilience App, TrackActive Me: Virtual Physio, PocketFriend Digital Therapy, ChatPal, Buoy Health, Chat Bot, Mediktor - Symptom Checker, Naturopathy Assistant, Ziver - Always On, MayaMD, NatHealth VA, OWL Cancer Survivor Platform, RiseUp: AI based CBT Therapy, Virtual Assistant: AI Friend, Sensely, Reflectly - Journal & AI Diary, ChatGPT, SophieBot AI, TheraTalk, WellWisher, MARCo: Mental Health Friend, AskFirst (formerly Ask NHS), Replika: My AI Friend, Elysai: Talk to AI Friends, Magnify Wellness, 7 Cups: Therapy & Support |
|  | Students | 4 (11.1) | Wysa: Mental Health Support, Buoy Health, ChatGPT, Laura |
|  | Patient | 1 (2.8) | Elena-The Medical Assistant |
|  | Therapy or physician | 2 (5.6) | Anesthesia Assistant, ChatGPT |
| **Response to Suicidal Thoughts** | | | |
| Responding to suicidal thoughts | Providing coherent responses | 16 (44.4) | Wysa: Mental Health Support, WingMan For Addiction, Woebot: Your Self-Care Expert, Meela - Birth support and more, Driven Resilience App, ChatPal, Mediktor - Symptom Checker, MayaMD, ChatGPT, MARCo: Mental Health Friend, Laura, AskFirst (formerly Ask NHS), Replika: My AI Friend, Elysai: Talk to AI Friends, Magnify Wellness, 7 Cups: Therapy & Support |

1. Allowing users to input free text without providing a response or does not have the ability to understand. [↑](#footnote-ref-1)
